# Features and management of osteoarthritis from the perspective of individuals with osteoarthritis: A systematic review of qualitative studies

**DOI:** 10.1016/j.ocarto.2025.100590

**Published:** 2025-02-25

**Authors:** Sylvain Mathieu, Alice Courties, Céline Mathy, Serge Perrot, Françoise Alliot Launois, Stanislas Moumbe, Nathan Foulquier, Jérémie Sellam, Rinie Geenen, the GO-PAIN network

**Affiliations:** a Department of Rheumatology, Hôpital Gabriel Montpied, 63000 Clermont-Ferrand, France; b Department of Rheumatology, Saint-Antoine Hospital, Assistance Publique – Hôpitaux de Paris (AP-HP), Sorbonne Université, Centre de Recherche Saint-Antoine, Inserm UMRS_938, 75012 Paris, France; c The Osteoarthritis Foundation, Boncelles, Belgium; d Pain Center, Inserm U987, Hôpital Cochin, University of Paris Cité, Paris, France; e Association Française de Lutte Antirhumatismale (AFLAR), Paris, France; f Patient Research Partner, Department of Rheumatology, Saint-Antoine Hospital, Assistance Publique – Hôpitaux de Paris (AP-HP), Sorbonne Université, Centre de Recherche, France; g LBAI, UMR1227, Univ Brest, Inserm, 9 Rue Felix Le Dantec, Brest, 29200, France; h Department of Psychology, Utrecht University, Heidelberglaan 1, 3584 CS Utrecht, the Netherlands; i Insrm U-1107, NeuroDol, Clermont-Ferrand, France

**Keywords:** Osteoarthritis, Pain, Patient-centered care, Qualitative research

## Abstract

**Objective:**

To enable person-centered care, considering beliefs, needs, and priorities of individuals with osteoarthritis (OA) is crucial. Nevertheless, concepts that they consider important are not fully recapitulated in assessment and care. The aim of this study was to clarify how individuals with OA conceive, experience, and manage their OA and pain.

**Design:**

A systematic literature review was conducted including qualitative studies (interviews, focus groups, open questionnaires) regardless of OA joint location. Verbatim quotations relating to OA and OA-related pain were collected and merged in codes. Themes and categories relating to these codes were defined.

**Results:**

The seven databases yielded a total of 9585 studies of which 79 qualitative studies were selected. Analysis of 667 verbatim quotations of 2009 participants led to 117 codes and 24 themes. Themes were grouped into 2 categories, ‘features’ and ‘management’. ‘Features’ encompassed experiences ranging from common challenges (e.g., adjust to reduced function) to high impact outcomes needing therapeutic attention (e.g., devastating pain). The ‘management’ category captured positive and negative conceptions associated with pharmacological and cognitive-behavioral self-management, psychoeducation, and interventions. Themes from both categories were classified into four domains: symptoms, functioning, psychological, and social. A fifth domain ‘disease’ was also used to categorize the themes under ‘features’.

**Conclusions:**

Several themes reported by OA individuals are hardly represented in current assessment and recommendations, e.g., fatigue, sleep disturbance, psychosocial impact, and effects on family and caregivers. The reviewed beliefs, needs, and priorities may support individualized screening, complement existing assessment instruments, and can help refine interventions and psychoeducational materials.

## 1 Introduction

Osteoarthritis (OA) is a leading cause of disability and one of the conditions contributing most to the global Burden of diseases [1]. Pain is the dominant symptom in OA and a major concern for patients [2]. Inadequate pain management negatively impacts physical function and mental well-being. Pain is considered a biopsychosocial phenomenon comprising multiple and mutually interacting biological, psychological and social factors which extend beyond joint damage to encompass central neurophysiological processes, physical (dis)ability, resilience and vulnerabilities (emotions, cognitions, lifestyle), social factors (work), sleep quality, and obesity [3]. To achieve effective assessment, psychoeducation and management, it is important to hear the voice of patients.

Recommendations consistently emphasize the importance of shared decision making and management of OA and pain tailored to the needs of each patient [[3], [4], [5]]. This requires consideration of many factors including patient's needs, preferences and priorities, individual pain characteristics (location, intensity, type, spread, and quality of pain), previous and ongoing pain treatments and their perceived efficacy, the extent of joint damage, and other pain-related factors including beliefs and emotions about pain and pain-related disability, social influences, sleep problems, and obesity [3]. A qualitative review concluded that many concepts important to individuals with hand OA are not fully represented in the most commonly used assessment instruments [6]. Other previous reviews of qualitative studies in OA focused on specific topics, such as barriers and facilitators of physical activity in knee and hip OA [7], attitudes of individuals with in knee or hip OA towards conservative management [8], and experiences of living with knee [9] or hip OA [10], but no study reviewed all the experiences of individuals with any kind of OA.

Thus, concepts that are important to individuals with OA are not fully represented in commonly used instruments and knowledge of beliefs, needs, and priorities of people with OA are important to be able to offer person-centered care. Therefore, to be able to formulate points to consider to improve assessment and management of OA, the aim of the current study was to clarify how individuals with any kind of OA conceive, experience, and manage their OA and pain.

## 2 Methods

This systematic literature review of qualitative studies was done in accordance with the methods for the thematic synthesis of qualitative research in systematic reviews [11]. The research protocol was registered on PROSPERO (CRD42024550601).

### 2.1 Literature search

We conducted a comprehensive search across 7 databases: MEDLINE via Pubmed, EMBASE, Scopus, Web of Science, PsycInfo, CINAHL, and the Cochrane library to identify all qualitative studies related to OA and OA-associated pain from inception through October 2023. The search strategy comprised terms relating to three key concepts: ‘osteoarthritis’, ‘pain’ and ‘qualitative research’. For each concept, keywords and Medical Subject Heading terms were combined. Detailed search equations are available in Supplementary Table S1.

### 2.2 Eligibility criteria

Included qualitative studies were interviews, focus groups, and questionnaire studies with open questions, regardless of joint location (knee, hip, hand, or other) with qualitative data (quotations) reflecting the points of view of individuals with OA. All the qualitative studies concerning patients with OA were included, whatever the affected joint(s). Studies using mixed samples of participants (e.g., with and without OA) were also included, but we only extracted quotations from patients with OA. For a study to be included, it was mandatory that at least one quotation should relate to pain, but we also collected any other quotation that might be associated with OA or pain, such as social or family life, work, fears, barriers, benefits, and expectations. Excluded were commentaries, protocols, editorials, case reports, studies concerning children, studies with a quantitative design, and studies with no full-text available. No comparator (e.g., a control group) was necessary in our study. Our search was restricted to original articles published in English or French. Review articles were excluded.

### 2.3 Study selection

Records were imported into Rayyan software [12]. Duplicates were removed. Potentially relevant articles were selected by screening titles, keywords, and abstracts, after which two investigators (SM and RG) independently reviewed full-texts. The other authors monitored the different steps of this selection, discussed discrepancies between the two investigators, confirmed included and excluded studies, and ensured that no studies of interest were missed. Final article selection was achieved through consensus among all investigators.

### 2.4 Data extraction

One investigator (SM) extracted all data from each study using a standardized data abstraction form. Extracted data were: the country where the study was conducted, the qualitative method (interview, focus group, questionnaire), the number of participants with OA, the joint location of OA, participants’ characteristics (mean age, percentage of women), and the pain intensity measured by the different scales. All quotations (words or sentences) related to the research question (i.e., conception, experience and management of OA and OA-related pain expressed by participants with OA) were recorded. Themes and sub-themes reported in the included studies were not extracted.

### 2.5 Data synthesis and report

We followed the procedure for thematic synthesis described by Thomas and Harden [13]. Verbatim quotations from individuals with OA were extracted from the articles. A code was attached to each quotation. Groupings of codes were subsequently organized into themes. Allocation of codes to themes was an iterative process in the research group until consensus was reached. We used domains and overarching categories to classify the themes. To remain as faithful as possible to the original quotations without interpretation, we did not extract codes or (sub)themes from the articles and refrained from extrapolating analytic themes.

The initial coding and grouping of codes into themes was completed by two researchers (SM and RG). The labeling and assignment of codes, themes, domains, and overarching categories was discussed through email and in video calls until consensus was reached between the researchers and patient research partners (SM, AC, CM, SP, FAL, SMb, JS, and RG).

### 2.6 Quality of the studies

The quality of the report of each included study was assessed using the consolidated criteria for reporting qualitative research (COREQ) checklist [14]. The COREQ checklist is a 32-item checklist for interviews and focus group designed to support authors in the reporting of qualitative studies. Five authors (SM, RG, AC, CM, and SP) graded part of the articles. They answered each question by ​« ​Yes ​» ​or ​« ​No ​» ​depending on the presence or absence of each criterion in the full-text article. The range of scores is 0–32 with higher scores representing better quality. The Critical Appraisal Skills Program (CASP) Qualitative Studies Checklist was used to assess the methodological quality of each included study. This checklist consists of 10 questions that cover rigor, methodology, credibility, and relevance [15]. The CASP analysis was done by one member of the project group (SM).

## 3 Results

### 3.1 Selected studies

**Fig. 1:**
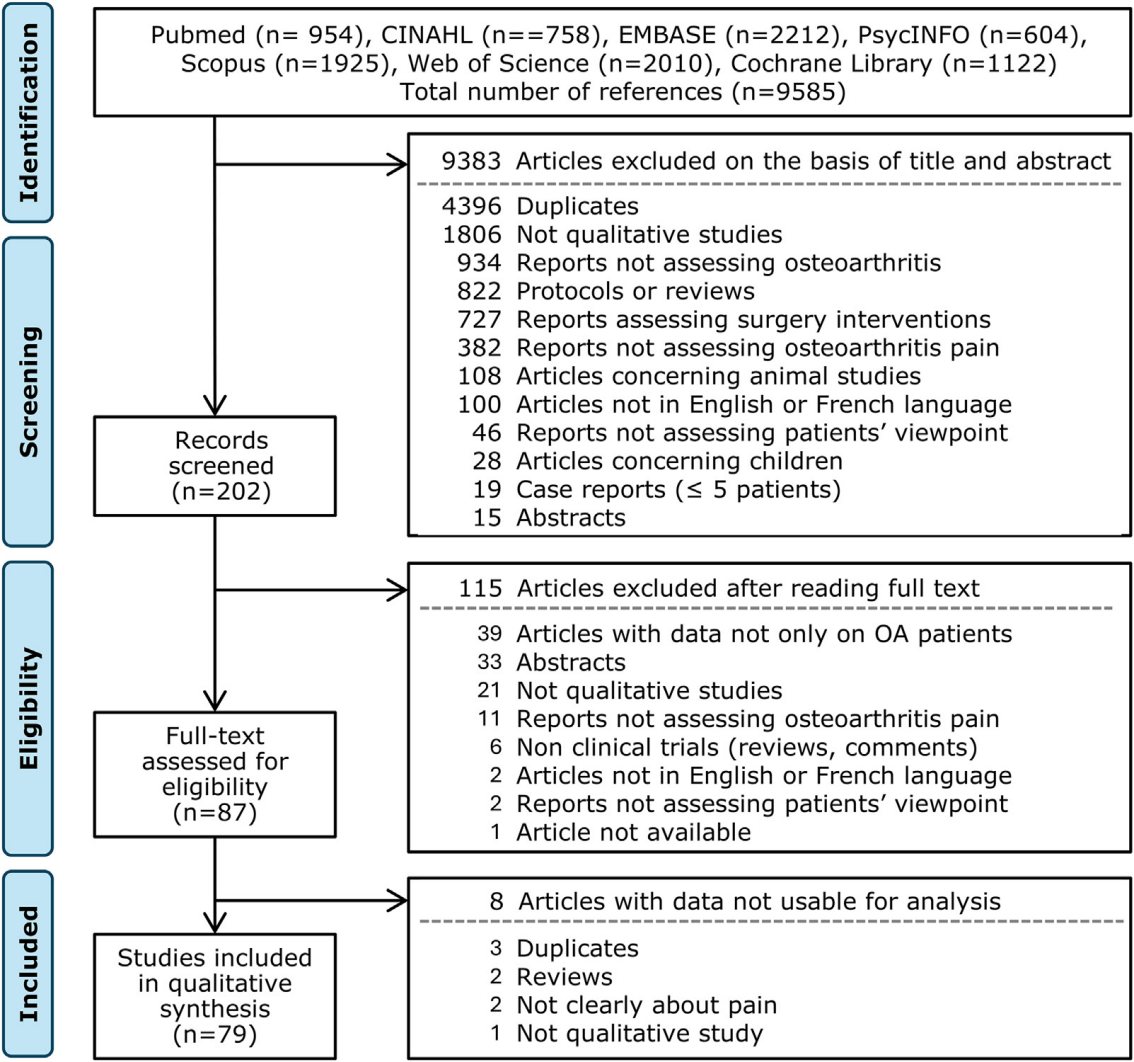
Flow diagram depicting the identification and selection of studies for inclusion in the review.

The seven databases yielded a total of 9585 citations with 79 studies meeting the eligibility criteria (Fig. 1 and Supplementary Table S2).

### 3.2 Characteristics of the included studies

The methods used in the 79 studies were 58 interviews, 14 focus groups, 4 questionnaires with open questions, and 3 not specified. The results of the quality assessments are given in the last column of Supplementary Table S2 (COREQ) and Supplementary Table S5 (CASP). Two studies were not assessed with the COREQ checklist because information was difficult to obtain from the dissertation and online-survey. The median COREQ score was 21 with a minimum of 8 and a maximum of 32. Most of the included studies had a good CASP methodological quality. Seven studies did not offer information about data analysis and collection or ethical issues.

### 3.3 Participant characteristics

The 79 studies included 2009 participants with OA. Gender data of 1814 participants were available representing 1232 (68 ​%) women. The mean age was 64 years, (range 20–92). The mean pain intensity was 4.5 on 0 to 10 Visual Analogue Scales (14 studies; 346 participants), 8.3 on the 0 to 10 pain scale of the Western Ontario and McMaster Universities Osteoarthritis Index (WOMAC, 3 studies, 86 participants), and 53.9 on the 0 to 100 pain scale of the Knee Injury and Osteoarthritis Outcome Score (KOOS, 3 studies, 134 participants). The studies were conducted across four continents: Europe (n ​= ​33), North America (n ​= ​24), Australia (n ​= ​14), and Asia (n ​= ​8).

### 3.4 The perspective of individuals with OA

**Fig. 2:**
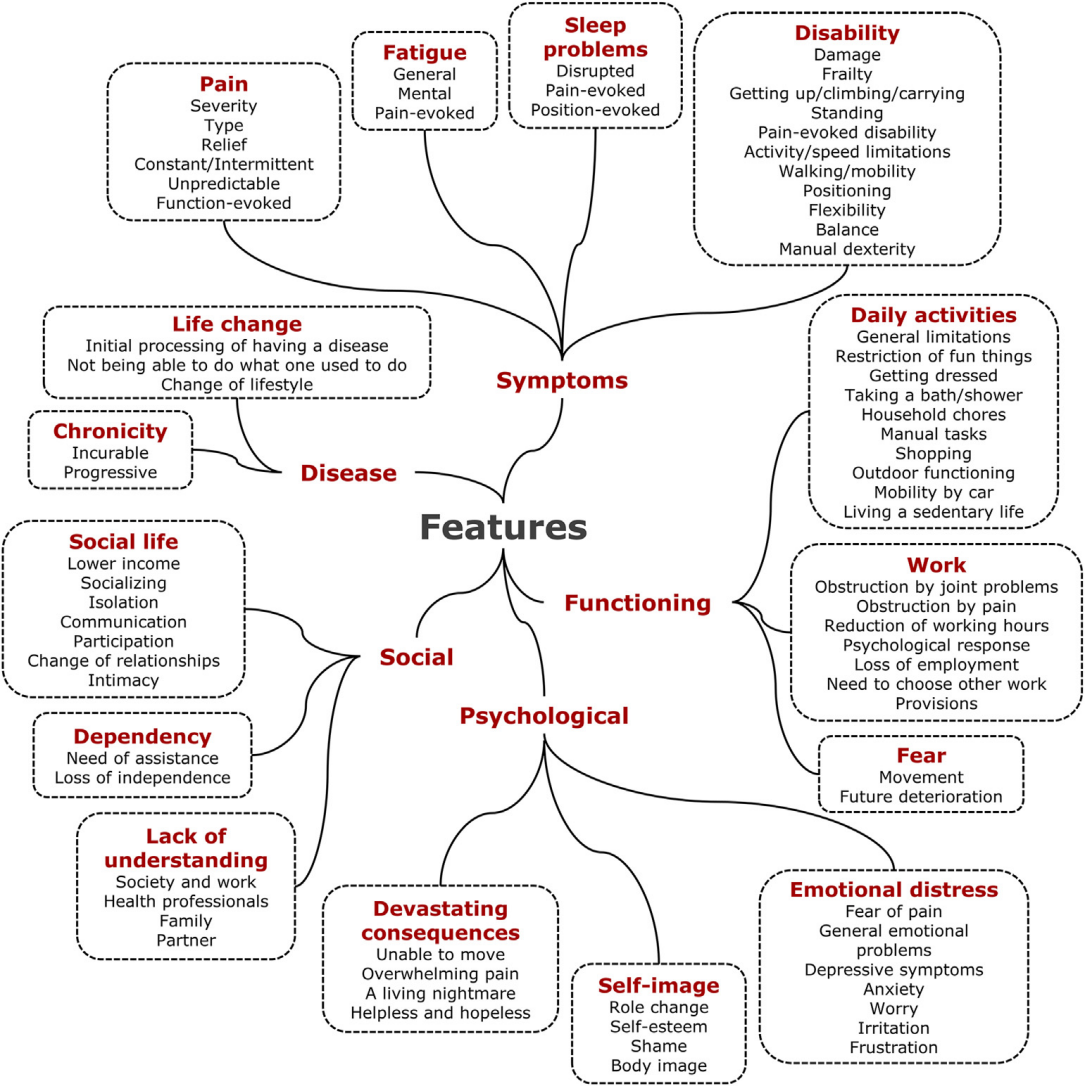
Overview of the 15 themes and 76 codes classified under features.

**Fig. 3:**
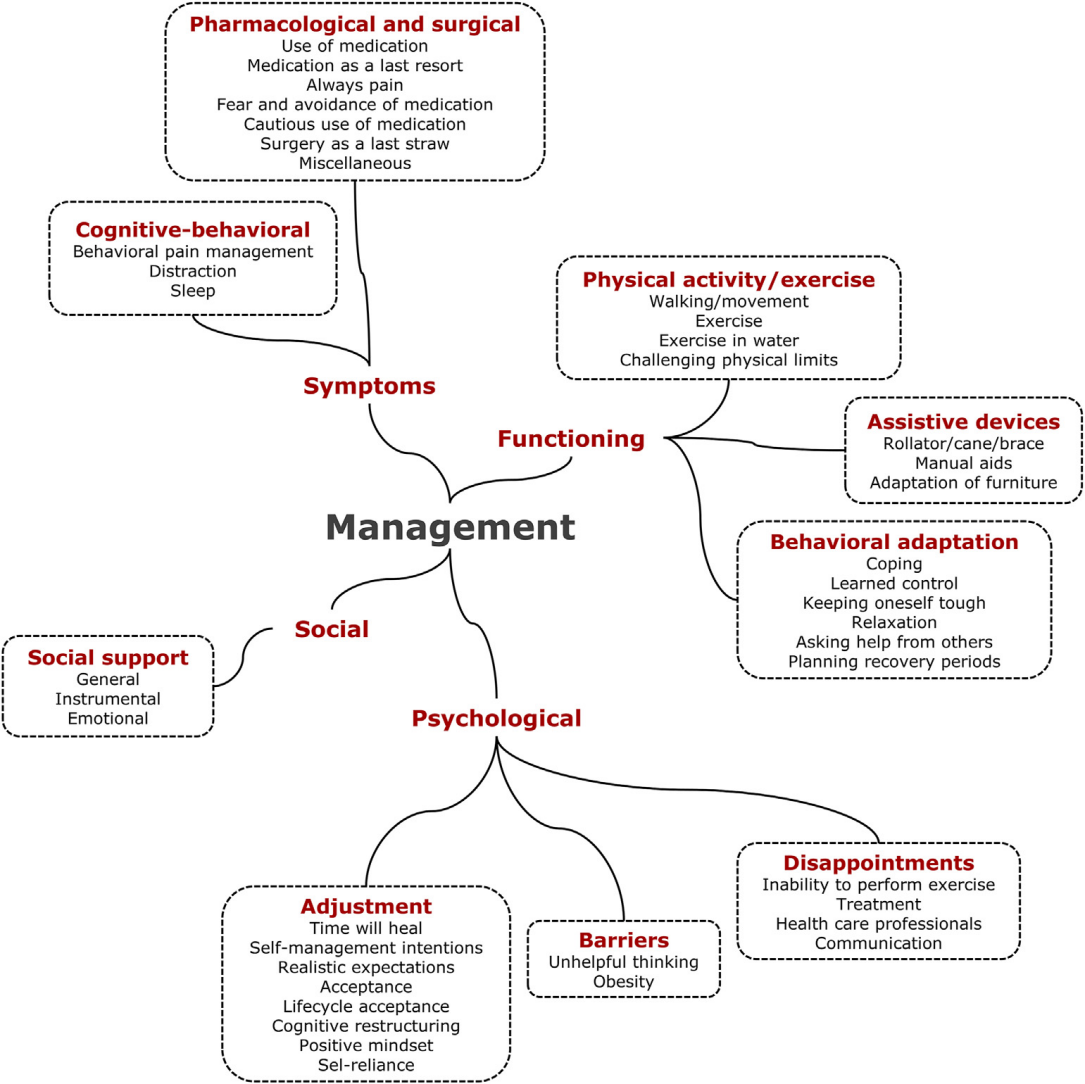
Overview of the 9 themes and 41 codes classified under management.

We obtained 667 verbatim quotations that were classified in 117 codes and 24 themes. The research group decided to split the themes in two main categories: features (Fig. 2) and management (Fig. 3) of OA. Example quotations are shown in Table 1 for features of OA and in Table 2 for management of OA. Supplementary Table S3 and Supplementary Table S4 show all quotations.

**Table 1:** Domains, themes, codes and example quotations of features of osteoarthritis from the perspective of individuals with osteoarthritis. All quotations are shown in Supplementary Table S3. In italics = domain; underlined = theme; in bold = code; “Example quotation”.

*Disease*
Chronicity
**Incurable “**It's incurable, if that's the word, and you've got to live with it and therefore manage it” [16] **Progressive** “Progressive: It's worse all the time” [17]
Life change
**Initial processing of having a disease** “It's just made me realize some things are going to get harder to do” [18] **Not being able to do what one used to do** “Oh, I won't be able to do that, and that … and I'd planned in retirement” [19] **Change of lifestyle** “Complete change in lifestyle is required …” [20]
*Symptoms*
Pain
**Severity** “Level pain is annoying” [21] **Type** “The pain is just an aching, dull pain that's always there” [22] **Relief** “No effective pain relief” [23] **Constant/Intermittent** “That it’s always sitting there in the background” [19] **Unpredictable** “Unsure when pain will come on” [24] **Function-evoked** “I have to be more careful than I used to, my hands are painful all the time when I use them” [6]
Fatigue
**General** “you're still fatigued when you wake up, you're still not there” [25] **Mental** “The mental fatigue is something much different. You just feel absolutely drained out and you can't focus” [25] **Pain-evoked** “knee pain cause fatigue which affected ability to work for a patient with little flexibility in work” [26]
Sleep problems
**Disrupted** “I can't sleep with it, what do I do?” [27] **Pain-evoked** “Sharp pain comes on at night, difficult to sleep” [24] **Position-evoked** “You don't even really sleep much at night because every time you go to turn, it takes an hour to find a comfortable position” [28]
Disability
**Damage** “It's really obvious I have no cushioning in that knee” [29] **Frailty** “I wasn't able to carry it by myself” [30] **Getting up/Climbing/Carrying** “I'm crippled, I can't get up” [31] **Standing** “When I tried to stand, my knees were so weak I was unable to stand up. Oh, such suffering” [32] **Pain-evoked disability** “Pain affects ability for physical activity” [23] **Activity/speed limitations** “I am limited in my activities” [33] **Walking/mobility** “not being able to walk properly [34] **Positioning** “Certain positions are difficult, and there's probably a way to accommodate it” [35] **Flexibility** “Two participants could no longer wear stockings or socks because they found it too painful to lean or lift their legs” [17] **Balance** “I can fall over very easily because I've got no balance [21] **Manual dexterity***“*Annoying, because sometimes, you know, you can't hardly grab things” [22]
*Functioning*
Daily activities
**General limitations** “It's the inability to, that I can't do things that I find is more debilitating than the actual pain” [36] **Restriction of fun things** “I get no enjoyment out of it [dancing] like I used to” [37] **Getting dressed** “When it was really bad […] I couldn't lift my legs to put socks on, shoes and like trousers and that” [38] **Taking a bath/shower** “Showering has become more difficult, because your hands just won't [19] **Household chores** “Impacted daily life, including the ability to engage in daily activities (e.g., housework, gardening, and grocery shopping)” [39] **Manual tasks** “When fueling I can't open the petrol cap anymore” [30] **Shopping** “Reduce frequency of shopping” [40] **Outdoor functioning** “Physical activity can also be really painful just in everyday activity, I take huge detours to avoid going up the stairs in the underground” [34] **Mobility by car** “it's difficult for me to just get out of the car” [41] **Living a sedentary life** “I don't do much. I sit down. It doesn't hurt a bit when I'm sitting or lying in bed” [27]
Work
**Obstruction by joint problems** “I worked in the goods department which was very tough for my fingers: you have to handle and to take hold of all the boxes” [30] **Obstruction by pain “**After working hard, I hurt” [42] **Reduction of working hours** “I've had to cut my hours down. I was finding it too much just working full-time” [43] **Psychological response** “I was missing work” [44] **Loss of employment** “I had to quit my job” [30] **Need to choose other work** “moving from running a family business restaurant to an office job that was more sedentary, stating “I had to give it up, I could hardly walk” [26] **Need of recovery during and after work** “Increased need for recovery during or at the end of work” [26] **Provisions** “Lack of health and safety policies within the workplace to assist workers” [26]
Fear
**Movement** “When I'm walking, I feel very insecure, I don't feel safe, very vulnerable” [36] **Future deterioration** “It worries me that one day I won't be able to do the things I can do today” [29]
*Psychological*
Emotional distress
**Fear of pain** “I'm frightened to go … I daren't just because of the pain in my back” [45] **General emotional problems** “Psychological problems, including mental and emotional problems, such as anxiety, fear, frustration, feeling worn out, anger, sadness” [6] **Depressive symptoms** “I feel sad, downhearted, depressed” [46] **Anxiety** More anxious about what's going on, sometimes waking up in “sheer anxiety.” [39] **Worry** “I am worried, I sometimes think that if it hurts when I do something it will cause even more damage” [47] **Irritation** “Easily get annoyed and argue on minor things with the family/loved ones” [40] **Frustration** “It's frustrating not being able to accomplish as much as I'd like to some days” [19]
Self-image
**Role change** “I used to be the custodian and I gave it up” [48] **Self-esteem** “I'm just useless, just because of a daft ankle” [37] **Shame** “I don't want them to think like, there she is, walking around everywhere when she's too sick to be at work” [49] **Body image** “I don't like limping… I think it don't look nice” [50]
Devastating consequences (fatalism)
**Unable to move** Can't do it. Can't do it anymore [24] **Overwhelming pain** “Wracking pain all over my body at once. It's devastating” [51] **A living nightmare** “You're already dealin’ with enough emotionally, mentally when your whole life has been flipped upside down and it's somethin’ you know is uncurable” [51] **Helpless and hopeless** “I feel helpless, loss of control” [46]
*Social*
Social life
**Lower income** “Careful with money—money was now spent only on essentials and holidays forfeited” [52] **Socializing** “It's had a terrific effect on the social side of things” [50] **Isolation** “We don't have many guests for dinner anymore” [30] **Communication** “Pain inhibits all the communication you have with people, your relationships” [53] **Participation** “I am unable to take part in social activities I want to” [46] **Change of relationships “**Relationships of friends that maybe has suffered the most” [25] **Intimacy** “Sexual activities must then be adapted, which can limit sexual health” [54]
Dependency
**Need of assistance** “I depend on my husband a lot more now” [24] **Loss of independence** “Just being able to do things on your own without asking for help. Uh, huh, I'd like to be a whole person” [27]
Lack of understanding
**Society and work** “I feel not being understood, other people do not understand what I am suffering” [46] **Health professionals** “Health professionals need to understand that it is very wearing and tiring, and that it changes your life” [55] **Family** “Osteoarthritic pain is real although families can't physically see or choose not to ‘see’ the pain” [51] **Partner** “My husband thinks that I am complaining all the time” [33]

**Table 2:** Domains, themes, codes and example quotations about management of osteoarthritis from the perspective of individuals with osteoarthritis. All quotations are shown in Supplementary Table S4. In italics = domain; underlined = theme; in bold = code; “Example quotation”.

*Symptoms*
Cognitive-behavioral
**Behavioral pain management** “I'll put under my knees and then I'll put a cold pack on them” [67] **Distraction** “I try not to even focus on it, so I certainly don't keep track of it” [68] **Sleep** “sometimes sleeping with a hot water bottle, using a knee brace during the day, and using emu oil” [39]
Pharmacological and surgical
**Use of medication** “I suppose I'd come home and take half a pill or full pill, and just stay home” [42] **Medication as a last resort** “Sometimes I can't control it, and then I go to the analgesics” [66] **Always pain** “Medicine helps some, but not much, I am constantly in pain” [60] **Fear and avoidance of medication** “I believe that if you take painkillers you'll become worse and get more pain in the end” [66] **Cautious use of medication** “I try not to take medication every day” [66] **Sleep medication** “I have to be competent, I have to be alert, I have to think, so I absolutely refuse to take anything in the daytime. But I take it at night because if I didn't, my body would never relax for me to actually fall asleep” [28] **Surgery as a last straw** “Knees were a small thing not to worry about or a funny joint that can be surgically replaced compared to ankle or back” [26] **Miscellaneous** “Adherence to pills: not consistent, not taking the meds the same time every day” [60]
*Functioning*
Physical activity/exercise
**Walking/movement** “I know that simply going for a walk every day is very good for me” [47] **Exercise** “Finding exercise helpful for pain relief” [69] **Exercise in water** “Water exercise: It's very beneficial because I really can move without hurting myself as opposed to doing land aerobics” [69] **Challenging physical limits** “people ask me why I am huffing and puffing. I don't stop until I have finished whatever I am doing, but afterwards; I am totally worn out” [70]
Assistive devices
**Rollator/cane/brace** “Assistive devices to walk out doors: I use a cane all the time” [17] **Manual aids** “I have had to put a strap to the accelerator handle so it is jammed when I mow the lawn because I can't keep it pressed during mowing” [30] **Adaptation of furniture** “it's quite deliberate that handles of the cupboard doors have been replaced with thick wooden handles providing enough space for all of my hand and also that they are not cold to hold” [30]
Behavioral adaptation
**Coping** “Cleaning the house now requires planning so that less effort will be required” [17] **Learned control** “I done dealt with it so long that I've adjusted myself to the pain” [71] **Keeping oneself tough** “Don't give up, keep going. Don't give up. Don't let it get on top of you” [18] **Relaxation** “I sit for a while, I relax, and that's it” [66] **Asking help from others** “my daughter helped me sell betel nuts and I helped look after her child” [41] **Planning recovery periods** “I try to limit the number of strenuous activities per week, and spread them out over the days of the week so I don't overdo it any one day and get the job done without aggravating my osteoarthritis” [68]
*Psychological*
Adjustment
**Time will heal** “Well, I can't do this right now. I have to wait” [51] **Self-management intentions** “I had made an agreement with myself to try knitting again but with thick knitting needles” [30] **Realistic expectations** “But you've got to adjust your life to what you can do”^60^ **Acceptance** “Oh look, I've had it for so long, I just, it's just part of life” [16] **Lifecycle acceptance** “I think that as you get older, you expect such things to occur … things you might have to accept” [72] **Cognitive restructuring** “It's got to be self. To me, I think a lot of it's got to be self. If you wanna manage that pain, it's got to be up here [mind]. I can do this. I'm gonna manage this” [69] **Positive mindset** “Keep a positive mind, live on that day” [73] **Self-reliance** “I was fairly confident that would give at least some benefit” [16]
Barriers
**Unhelpful thinking** “When I start thinking to myself I'm fed up, I've got to stop that and think positive” [18] **Obesity** “Person's self-responsibility regarding “controlling” knee pain [WG] by “keeping weight down” [RN], recognizing the consequence of carrying “extra weight” [JG] to the knee joint as “taking a toll on the body” [26]
Disappointments
**Inability to perform exercise** “Exercise makes knee pain worse” [60] **Treatment** “Do not expect that OA treatments will significantly help my symptoms” [62] **Health care professionals** “There's nobody that appears to be an expert in osteoarthritis” [53] **Communication** “He was more interested in taking pictures of my knee than in examining it” [20]
*Social*
Social support
**General** “I'm alright if my husband's with me and I can hold his arm” [36] **Instrumental** “hire people, help & support from family members” [40] **Emotional** “Lots of bonding, lots of support, lots of strength from them, especially from my parents” [66]

### 3.5 Category 1: Features of OA

The category encompassing 15 themes and 76 codes related to ‘features’ (Fig. 2, Table 1, Supplementary Table S3) captures a range of experiences associated with OA, varying from common challenges to high impact outcomes needing therapeutic attention. Besides showing differences *between* individuals, these features may also fluctuate across the course of OA *within* persons. The features category was subdivided into five domains: 1. disease, 2. Symptoms, 3. Functioning, 4. Psychological, and 5. Social.

*Domain 1: Disease*. This domain included two themes: *chronicity* and *life change*. The ‘chronicity’ theme comprised beliefs that OA was *incurable*, e.g., “It's incurable, if that's the word, and you've got to live with it and therefore manage it” [16], “you can't change osteoarthritis” [29] and *progressive*, e.g., “It's worse all the time” [17]. The ‘life change’ theme included three codes: *initial processing of having a disease, not being able to do what one used to do*, and *change of life style*. Quotations reflected that experiences and anticipations of the change of life may vary from small, e.g., “My life is somewhat restrictive” [33], to large, e.g., “complete change in lifestyle is required” [17]. Several patients reported an awareness of the need for a change of life, e.g., “It's just made me realize some things are going to get harder to do” [18], and “Oh, I won't be able to do that, and that … and I'd planned in retirement” [19].

*Domain 2: Symptoms*. Within the domain ‘symptoms’, 23 codes were identified across four themes: pain, fatigue, sleep, and disability. The codes reflect variations in how individuals with OA experience the impact of symptoms on their lives. For instance, within the theme ‘pain’, quotations included under the code *severity* were horrific [37] or annoying [21] as well as minimal pain [36], and the code *relief* reflected both alleviation and lack of alleviation [23,56]. The codes may also hint to further assessment and management that is needed, for instance, whether the perceived *type* of pain is sharp [17] or widespread [57], which may indicate whether the pain is nociceptive, neuropathic, or nociplastic [58]. Codes such as *function-evoked pain, pain-evoked fatigue, pain-evoked sleep*, and *position-evoked sleep* underscore that some individuals with OA not only report the type or severity of a specific symptom but also the perceived triggers, e.g., feeling pain when climbing stairs [32], and perceiving pain as the cause of fatigue [59] or sleep problems [28]. Different aspects of ‘fatigue’ were reported, e.g., that the fatigue was *generalized*, reflecting complete exhaustion [25], or *mental* reflecting concentration or motivational problems [25,60]. ‘Disability’ was a particularly large theme, encompassing 11 codes related to specific types of (dys)function of the body: *damage, frailty, getting up/climbing/carrying, standing, pain-evoked disability, activity/speed limitations, walking/mobility, positioning, flexibility, balance*, and *manual dexterity*. Some dysfunctions typically involved OA in lower extremity joints, as reflected in the following quotations: “I'm crippled, I can't get up” [31], “When I tried to stand, my knees were so weak I was unable to stand up” [32]. Other quotations reflected disability due to OA of the hand or thumb, e.g., “I couldn't hold a hammer. I couldn't grip, the tools” [22]. Damage and pain were suggested to play a major role in disability: “Just doing things, you can feel it clicking and it rubs against one another” [61], “Pain affects ability for physical activity [23].

*Domain 3: Functioning*. This domain encompassed three themes: daily activities, work, and fear. Ten codes were grouped in the theme ‘daily activities’. Most codes refer to common daily activities that are obstructed, e.g., *general limitations. getting dressed, taking a bath/shower, household chores, manual tasks, shopping, outdoor functioning, mobility by car*, and *living a sedentary life*. However, the code *restriction of fun things* reflects that these obstructions in functioning are a threat to quality of life, e.g., “I cannot do things I enjoyed doing” [46] and “It stops me playing with the grandchildren” [21]. The theme ‘fear’ included two codes that could also have been included in the psychological domain: fear of *movement* and of *future deterioration*. Eight codes were included in the theme functioning at ‘work.’ Individuals with OA reported that work was *obstructed by joint problems* and *obstructed by pain*, e.g., “I had to retire at 62½ because I couldn't do my job” [62]. Consequences were a *reduction of working hours*, *loss of employment*, and a *psychological response*, e.g., missing work and loss of self esteem [44,50]. Participants in the studies also emphasized the need to adapt current work, “I've had to cut my hours down” [21,43], and the wish or necessity to choose other work, “Has to change work tasks, has to change career plans” [6].

*Domain 4: Psychological*. This domain comprised 15 codes included in 3 themes: emotional distress, self-image, and devastating consequences. These codes and quotations mostly highlight the negative influence of OA on emotional well-being and self-image with ‘devastating consequences’ reflecting the most overwhelming impact [51,63]. Three codes within the theme ‘emotional distress’ predominantly included features that patients associated with OA pain and disability: *fear of pain, worry, and frustration*, e.g., “I am not very worried about my knee collapsing on me, but I'm a little worried that the condition of my knee will continue to deteriorate” [63] and “It's quite depressing cos if I'm planning to do something and then I sort of can't because it's started to hurt [36]. The other features of emotional distress were sometimes attributed to pain and disability, but were often mentioned by patients without a connection to specific problems of OA: *general emotional problems, depressive symptoms, anxiety*, and *irritation*, e.g., “I tend to get depressed or upset with myself” [25], “More anxious about what's going on, sometimes waking up in sheer anxiety” (O'Brien 2023) [39], and “I become irritable, impatient, and I lose temper easily” [46]. The ‘self-image’ theme included four codes: *role change, shame, self-esteem*, and *body-image*. These codes illustrate that the deformation of the body and loss of function may have consequences for how individuals with OA appreciate themselves. The ‘devastating consequences’ theme included four codes: *unable to move, overwhelming pain, a living nightmare*, and *helpless and hopeless*. Quotations within this theme show that some individuals with OA suffer excessively, e.g., “The biggest thing for me is that [the pain] is gonna get worse and worse” [63], “it's laborious enough just livin” [51], “Wracking pain all over my body at once. It's devastating” [51], “Living with arthritis, it's a living nightmare” [64].

*Domain 5: Social*. This domain comprised 3 themes: social life, dependency, and lack of understanding. The 7 codes listed under the theme ‘social life’ reflect social-economic, interpersonal, relational, and societal consequences. Individuals with OA reported a *lower income*, often work-related (e.g., Ref. [32]), and problems with *socializing* (e.g., Ref. [50]), *communication* (e.g., Ref. [53]), and *participation* (e.g., Refs. [6,49]). Consequences for the *intimate relationship* were also observed, e.g., “Could not sleep at night because of her OA pain” [65] and “Sexual activities must then be adapted, which can limit sexual health” [54]. Two codes were categorized within the theme ‘dependency’: *need of assistance* and *loss of independence*, e.g., “I have to depend on my husband to be able to do something” [66], and “Just being able to do things on your own without asking for help. Uh, huh, I'd like to be a whole person” (Maly 2007) [27]. Probably due to the invisibility of symptoms such as pain and fatigue, several patients experienced ‘lack of understanding’ by *society and work, health professionals, family*, or the *partner*. A request of one participant was that “health professionals need to understand that it is very wearing and tiring, and that it changes your life” [55].

### 3.6 Category 2: Management of OA

The category with 9 themes and 41 codes related to ‘management’ reflects experiences with self-management, psychoeducation, and interventions by health care professionals (Fig. 3, Table 2, Supplementary Table S4). We distinguished four of the five domains that were also used in the group with ‘features’: 1. Symptoms, 2. Functioning, 3. Psychological, and 4. Social.

*Domain 1: Symptoms*. With respect to management of symptoms (Table 2), participants shared their experiences with ‘cognitive-behavioral’ and ‘pharmacological and surgical’ management (2 themes). Most quotations within the three codes of the ‘cognitive-behavioral’ management theme reflected positive ways of coping with symptoms: *behavioral pain management* (“Compensate using the better knee” [26]), *distraction* (“I try not to even focus on it” [68]) and *sleep* (“I might jump in the bath in the middle of the night … and then I'll be able to sleep another couple of hours” [48]). In contrast, the quotations included in the 8 codes of the ‘pharmacological and surgical’ management theme also often included negative beliefs, fears, and reluctances about medical treatments. The codes were: *use of medication, medication as a last resort, always pain, fear and avoidance of medication, cautious use of medication, sleep medication, surgery as a last straw*, and *miscellaneous*. Some cautiously used medication, reported medication to be insufficient, or to cause fear, e.g., “Only take a pill when I am in terrible pain, otherwise I am against drugs” [20], “I try not to take medication every day” [66], “Medicine helps some, but not much” [60], “I'm also very afraid of taking medications” [35]. However, pharmacological and surgical interventions were also considered an opportunity if other efforts to manage symptoms failed: “Knees can be surgically replaced compared to ankle or back” [26], “Without medication I would be really limited, maybe 60 ​%” [27].

*Domain 2. Functioning*. This domain included thirteen codes categorized in three themes: physical activity/exercise, assistive devices, and behavioral adaptation. Three of the four codes of the theme ‘activity/exercise’ predominantly reflected benefits from *walking/movement, exercise*, and *exercise in water*: “I know that simply going for a walk every day is very good for me” [47], “Exercising to manage the knee pain” [74], “Swimming reduced my pain” [75]. Several quotations reflected the perceived benefits of not exceeding limits, but quotations under the fourth code reflected that some people *challenge their physical limits*. The theme ‘assistive devices’ included three codes: *rollator/cane/braces, manual aids*, and *adaptation of furniture*. The quotations of patients generally reflect that assistive devices are helpful and reassuring, e.g., “I know I can balance well [with a walker] because I am worried about doing more harm if I fell” [61]. The third functioning theme, ‘behavioral adaptation’, included six codes: *coping, learned control, keeping oneself though, relaxation, asking help from others*, and *planning recovery periods*. Virtually all quotations reflect beneficial experiences with change of behavior that helped to function better. Example quotations of each code are: “I just try to take control of it myself. Then with assistance from my family” [51], “I actually have managed to live with the pain” [72], “Our grandparents had osteoarthritis. They never complained” [59], “I sit for a while, I relax, and that's it” [66], “My daughter helped me sell betel nuts and I helped look after her child” [41], “I try to limit the number of strenuous activities per week, and spread them out over the days of the week so I don't overdo it any one day” [68]. Few quotations reported whether management experiences were self-invented solutions, suggestions from peers, psychoeducation, or due to professional support from physicians, physiotherapists, occupational therapists, or psychologists.

*Domain 3, Psychological*. This management domain included 14 codes within 3 themes: adjustment, barriers, and disappointments. The theme ‘adjustment’ mostly included cognitive-behavioral adaptations helping individuals to deal with the OA and its consequences. The following eight codes were included: *time to heal, self-management intentions, realistic expectations, acceptance, lifecycle acceptance, cognitive restructuring, positive mindset*, and *self-reliance*. Even when everything has been tried to relieve symptoms and adapt behavior, adverse effects of OA remain. This theme mainly reflects that people with OA then choose cognitive ways of dealing with these consequences by *accepting* that the disease is “part of the ageing process” [67], and by striving for a *positive mindset*, e.g., “I just cope and enjoy the company and the food and the peoples” [76]. Also *cognitive restructuring* or influencing one's thoughts were mentioned as a type of adjustment, e.g., “It is always there, but it isn't always severe” [77]. However, psychological management could also be obstructed by *unhelpful thinking* and *obesity*, the two codes included in the second theme, ‘barriers’. The third theme, ‘disappointments’, included four codes: *inability to perform exercise, treatment, health care professionals*, and *communication*. Examples reflecting dissatisfaction listed under each code are, respectively: “Exercise makes knee pain worse” [60], “There is nothing that can be done about the OA; therefore, I do nothing” [78], “I don't think [my family doctor] knew what to do” [62], “I asked my questions and they told me that they didn't want to spend time on them” [79].

*Domain 4: Social*. This management domain included one theme, ‘social support’, with three codes: *general, instrumental*, and *emotional*. It showed the perceived benefits of general support, e.g., “help & support from family members” [40], instrumental support, e.g., “letting my family help me even though I am not happy” [80]), and emotional support, e.g., “it was good for me that someone said, ‘I see that it is painful.’ I felt I got help, really” [81].

## 4 Discussion

Our systematic review of 79 studies yielded 667 verbatim quotations that were categorized in 117 codes, 24 themes, and 2 main categories: ‘features’ and ‘management’. The ‘features’ category included experiences associated with OA that varied from common challenges in daily life to high impact outcomes requiring therapeutic attention. The ‘management’ category comprised experiences with self-management, psychoeducation, and interventions by health care professionals. Four domains were used to group the themes of both ‘features’ and ‘management’: symptoms, functioning, psychological, and social. Additionally, a fifth domain ‘disease’ was used to categorize the themes under ‘features’.

Previous review studies also adopted a biopsychosocial framework, but were focused on only knee OA [9], or hip OA [10] or both [7,8]. One review focused on barriers and facilitators regarding physical activity and exercise [7]; many barriers and facilitators are also represented in our review. The review also explored differences in barriers and facilitators between lifestyle physical activity and uptake and maintenance of physical activity [7], which are not represented in our review. Several themes of the other review studies showed overlap with the themes of our study, but there were differences as well. Studies did not or hardly report what kind of assessments patients wanted [[8], [9], [10]], patients’ perceptions of interventions they received [9], fatigue [9,10], sleep [8,9], detailed consequences for work [[8], [9], [10]], the impact on partners [[8], [9], [10]], and specific functional impairments such as problems with multi-joint or upper limb OA [[8], [9], [10]]. All reviews yielded implications for management of OA. In contrast to these previous reviews, our review offered an encompassing overview including any type of OA independent of the affected joints and also formulates points to consider to improve assessment.

Features of OA differ *between* individuals and may, across the course of OA, also vary *within* persons. More than 90 tools have been used to assess pain and physical function in patients with OA [82]. Besides the Intermittent and Constant Osteoarthritis Pain questionnaire to assess pain [83], commonly used self-report questionnaires to measure both pain and function are the WOMAC and KOOS [84,85]. WOMAC also includes self-reports of stiffness and KOOS assesses the impact of OA on quality of life. In our review, disability emerged as a large category included under ‘features.’ Stiffness appears to be experienced by patients as an obstructive aspect of functioning rather than as a symptom. Fear of movement and future deterioration surfaced as issues that could exacerbate functional limitations. Such fears were shown to be associated with underestimation of self-report functional abilities in relation to actual walking ability [86], and warrant close attention in clinical management to prevent further disability.

Outcomes of the OMERACT-OARSI Core Domain Set, which were deemed essential by both patients and other stakeholders (health professionals, researchers, industry) include pain, physical function, quality of life, patient's global assessment of the target joint, and (if indicated) joint structure [87]. These aspects of OA, especially pain and several themes of impaired physical function that are also shown to be important in our review, are represented in commonly used questionnaires. However, several outcomes were designated as ‘optional’ or ‘research agenda’ outcomes by the OMERACT-OARSI group despite being considered important by patients; that is, more patients than other stakeholders emphasized the importance of psychosocial impact (83 ​% votes from patients vs. 61 ​% votes from other stakeholders), sleep (88 ​% vs. 57 ​%), cognitive function (71 ​% vs. 20 ​%), fatigue (67 ​% vs. 23 ​%), or effects on the family/caregiver (52 ​% vs. 11 ​%) [87]. Our review corroborates that patients consider these outcomes important. For example, the ‘symptom’ domain highlighted fatigue and sleep problems besides pain and disability. Severe fatigue occurs in 1 out of every 3 individuals with OA [88]. As in inflammatory rheumatic diseases [89], this warrants regular assessment in clinical practice. Besides fatigue, other themes should be addressed in the care of patients with OA, such as psychosocial impact (emotional distress, self-image, devastating consequences), sleep disturbances, and effects on the family/caregiver (social life, dependency). Although these themes are indicated to be a prevalent burden in OA, they are not or only briefly mentioned in existing recommendations [4,5], perhaps because these themes are considered characteristic for virtually any chronic disease.

Our review indicated that effects of OA on work participation and working life duration are substantial, similar to previous studies [90]. The EULAR recommendations [5], but not OARSI guidelines [4], advocate that people with OA should be offered timely advice on modifiable work-related factors. Supporting individuals with OA in achieving healthy and sustainable work participation requires collective action from the person with OA, health care workers, colleagues, employers, work agencies, and politicians [91].

Quotations in the ‘management’ category referred to experiences of any kind of management, mostly without naming the source of management, e.g., self-management, social support, psychoeducation, and telehealth or face-to-face interventions. The ‘cognitive-behavioral management’ theme especially reflected self-management, while the ‘pharmacological and surgical management’ theme mostly reflected opinions and beliefs about the use of medication that varied from being positive about the effects of medication to cautious use, fear, and avoidance of medication. Requirement for joint replacement was reported by some people with OA as inevitable and a failure by others, as previously reported [8]. This impression of failure by patients with OA can be a realistic conception of the situation. In general, part of the preparation of any intervention is communication, explanation and exchange between the patient and the health professional. Negative beliefs can result in abandonment of treatment, while positive beliefs are associated with increased engagement in self-management and lifestyle-based interventions [92]. The reviewed quotations reflect how important it is that healthcare professionals listen to the patients and provide evidence-based and realistic information about the benefits and potential risks of specific medications. The categories with functional, psychological, and social management predominantly reflected the importance of self-management skills. Many quotations reflect that quite a few individuals with OA are doing well and know how to cope with OA and its consequences. The quotations suggest that individuals with OA could also learn from peers about how to adapt to adversities of OA. Nevertheless, several quotations reflect that some persons did not believe anymore that they were able to help themselves or could be helped by others. This indicates that there is a subgroup of individuals with OA that needs more intensive care, because they do not benefit from psychoeducational materials and current care.

Many participants with OA reported the benefits of physical activity on pain. Conversely, others experienced an increase of pain after physical activity and exercise and feared that it would accelerate the progression of their joint damage. In a previous review it was concluded that it was necessary to break down barriers and promote facilitators of physical activity [7]. These facilitators can include adaptation of a physical activity intervention to the patient's level. While patient education and exercise may be effective for many patients, our data illustrate that what holds for the majority of the group does not hold for everyone and that, therefore, alternative, individualized approaches are indicated for part of the patients.

Themes such as uncertainty about the cause of OA, having intense pain while the disease cannot be seen, and the unpredictable nature of pain and progression of OA are elements that often recur in patients' narratives. Taking into account the patients' viewpoint is essential in OA management. Perhaps our qualitative findings suggest that, from the patient's point of view, pharmacological treatments occupy a minority position in the management process, and are distrusted by some patients. Some quotations reflected that surgery is seen as a last straw solution. However, current guidelines unanimously discourage arthroscopy [93], and mixed results are reported regarding total joint replacement [94,95]. Although total joint replacement could be an option in selected patients with severe osteoarthritis [94], patients should be informed that surgery is not a cure-all solution. An unfavorable pain outcome has been reported in about 9 ​% after hip replacement and 20 ​% of patients with knee prosthesis [95]. Including these themes in education of health professionals could guide shared decision-making with the patient.

Therapeutic patient education is a structured person-centered learning process that supports individuals living with chronic conditions to self-manage their own health by drawing on their own resources, supported by their careers and families [96]. The overview of features and management issues given in our systematic overview including beliefs, experiences, barriers, and facilitators expressed by patients should be considered to enhance self-help materials and interventions. Our review did not yield information about the mode of management, such as self-management, social support, psychoeducation, telehealth, and face-to-face interventions. Tailoring of care to patient's needs and preferences is indicated to be important and in line with literature [[3], [4], [5],^89^,[97], [98], [99]] and with the opinions expressed by several respondents in our study, namely: that the tools and treatments proposed are not necessarily adapted to their daily lives and feelings, that patient-physician discrepancies regarding management exist, and that any recommendations on management of chronic diseases management should emphasize the need to consider patient perspectives and shared decision-making.

Both the OARSI and EULAR sets of recommendations/guidelines suggested that themes that are indicated by individual patients could or should also be integrated in care [4,5]. However, there is no evidence about superiority of individualized approaches over care that all patients should receive. Moreover, several themes that were considered important by patients have hardly been included in treatment recommendations, which might be due to lack of randomized controlled trials that investigated their effects. To support an individualized approach and shared decision making, Table 3 shows points to consider in assessment, psychoeducation, and management of OA motivated by the perspectives of individuals with OA. These points were prepared by the authors and discussed and validated by the entire Going Inside Osteoarthritis-Related Pain working group, which included a patient with OA and a representative of an OA patients’ association. Overall, these points to consider reflect that it is important in the management of patients with OA that they are involved in every stage of the process, as their expectations, needs, and priorities are not necessarily those anticipated by healthcare professionals.

**Table 3:** Points to consider in assessment, psychoeducation, and management of OA motivated by the perspectives of individuals with OA.

• Health care professionals should be prepared to support self-management and to offer psychoeducation or -if indicated- therapy, or to refer to other professionals. • To counter unrealistic beliefs about the progression of OA and anticipated devastating consequences, patient education should include realistic scenarios about the likelihood of several courses of OA. • Fatigue, sleep, psychological and social impact, and effects on family and caregivers should be part of assessment, care, and efficacy studies. • Information about modifiable work-related factors and, where appropriate, referral for expert advice should be offered. • It is important to listen to and take account of patient’ opinions and beliefs about medication and provide realistic and evidence-based information on the benefits and potential risks in line with existing recommendations. • Many individuals with OA acknowledge the importance of functional, psychological, and social self-management skills to deal with the consequences of OA. This suggests that, besides through professional care, individuals with OA can learn from each other how to adapt to adversities of OA and pain. • Individuals with OA who do not benefit from psychoeducational materials and current care and are helpless and hopeless, may need intensive psychological care. e.g., fixed consultations by the primary health care professional. Research should evaluate effects of therapy in this specific group. • Given the variability in disease status, personal and social factors, OA management should be individualized. Research should examine whether individualized approaches in care are superior to care that all patients receive. • It is mandatory to include the perspectives of patients with OA in the development of self-help materials for patients and education materials of health care professionals.

Our systematic review has limitations. The first one is related to the method we chose. The extraction of verbatims by a single investigator is a limitation of our study. However, this stage of data extraction solely involved the copying of all verbatims of patients that were reported in the studies, which limits the risk of error and subjective interpretation. The following steps in the coding and analysis of results were carried out by two investigators, with a discussion and validation by the rest of the team. We also followed a bottom-up procedure starting with allocating quotations to codes, allocating the codes to themes and grouping themes in categories. This procedure is by definition subjective, and our choice of codes and themes based on verbatims is open to debate. That's why each step was discussed and validated by consensus by the team that included patients, rheumatologists, psychologists, and a pain expert. Second, we aimed to give an encompassing overview of experiences of patients without interpretations of researchers. To that aim, we extracted the verbatims expressed by patients and not the themes or sub-themes chosen by authors. We assumed that the most exemplary verbatims were reported in the studies. However, the choice for verbatims that were reported will to a certain extend still be influenced by interpretations of the authors. Third, we cannot fully exclude that with the search terms we used, some studies were missed. Finally, we found studies mainly in Europe, North America and Australia, a few in Asia, and none in Africa or South America. Also, the exclusion of non-English and non-French language articles limits the generalizability to predominantly richer counties, and perhaps within these countries the middle and higher classes, who have access to at least a mediocre and often a good health care system. Therefore, our findings do not generalize to people from other cultures with other health care systems who may have different perceptions of OA and OA-related pain.

In conclusion, this review highlights considerable individual variability in symptoms, functioning, psychological and social variables, ranging from devastating impact to high adaptation. Several themes reported by patients are hardly represented in current assessment and management recommendations, e.g., fatigue, sleep disturbance, psychosocial impact, and effects on family and caregivers. The current encompassing overview of beliefs, needs, priorities and other individual differences may support individualized screening, complement existing assessment instruments, and can help refine interventions and psychoeducational and self-help materials.

## Author contributions

*Study conception and design*: Courties, Geenen, Mathieu, Mathy, Perrot, and Sellam.

*Acquisition of data*: Geenen, Mathieu.

*Analysis and interpretation of data*: All authors.

*Drafting the work or reviewing it critically for important intellectual content*: All authors.

*Final approval of the submitted version*: All authors.

*Accountability agreement*: All authors are accountable for all aspects of the work in ensuring that questions related to the accuracy or integrity of any part of the work are appropriately investigated and resolved.

## Ethical approval information

Ethical approval was not required for this study. It is a systematic review of studies for which the original investigators, who included research participants, will have sought approval from an Ethics Committee or Institutional Review Board.

## Data availability

No data were generated or analyzed for this study. Details on methodology are included in the article and supplemental data. An Excel file with quotations is available by request to the corresponding author.

## Funding

This project is initiated by the Going Inside Osteoarthritis-Related Pain working group. Two meetings of this working group were funded by NEURON, the “Network of European Funding for Neuroscience Research” established under the ERA-NET scheme of the European Commission (www.neuron-eranet.eu, NEURON-NL42) and ANR (Agence Nationale de la Recherche). The funder had no role in the design and conduct of the study; collection, management, analysis, and interpretation of the data; preparation, review, or approval of the manuscript; and decision to submit the manuscript for publication.

## Declaration of competing interest

S.M. declares that he received fees for presentations from Tilman; and support to attend meetings from Biogen, Lilly, Amgen and MSD.

A.C. declares that she received fees for presentations, manuscript writing, or educational events from Pfizer, UCB, Abbvie, Lilly, and Jansen; and support to attend meetings from Galapagos, Pfizer, BMS, UCB, MSD, Biogen, Novartis, and Janssen.

C.M. declares that she received fees for consulting and presentations from Expanscience.

J.S. declares that he received personal fees from MSD, Pfizer, Abbvie, Fresenius Kabi, Grünenthal, BMS, Lilly, Novartis, AstraZeneca, UCB and Janssen, and research grants from Pfizer.

R.G. declares that he received a consulting fee for a presentation from Pfizer.

N.F., S.M. and F. AL declare that they have no competing interest.
