# Supplementary material for: Features and management of osteoarthritis from the perspective of individuals with osteoarthritis: A systematic review of qualitative studies
Source: Osteoarthr Cartil Open. 2025 Feb 25;7(2):100590. doi: 10.1016/j.ocarto.2025.100590 (PMC12005280; doi:10.1016/j.ocarto.2025.100590)
Supplement: Multimedia component 1 [file mmc1.docx]

**Online supplementary file**

This online supplementary file is part of the article “Features and management of osteoarthritis from the perspective of people with osteoarthritis: a systematic review of qualitative studies” by Sylvain Mathieu, Alice Courties, Céline Mathy, Serge Perrot, Françoise Alliot Launois, Stanislas Moumbe, Nathan Foulquier, Jérémie Sellam, and Rinie Geenen, on behalf of the GO-PAIN network*

*Going Inside Osteoarthritis-Related Pain (GO-PAIN) network members: Françoise Alliot-Launois, Nadine Attal, Francis Berenbaum, Marie Binvignat, Philip Conaghan, Alice Courties, Niels Eijkelkamp, Camille Fauchon, Rinie Geenen, Ida K Haugen, Yves Henrotin, Kalle Kisand, Margreet Kloppenburg, Eva Kosek, Liisa Kuhi, Sylvain Mathieu, Céline Mathy, Ali Mobasheri, Stanislas Moumbe Talla, Patrick Omoumi, Serge Perrot, Roland Peyron, Simo Saarakkala, Alain Saraux, Hans-Georg Schaible, Jérémie Sellam

**Contents**

| **Table S1**. Search equations | 2-3 |
| --- | --- |
| **Table S2**. The 79 included studies | 4-8 |
| **Table S3**. Overview of features of osteoarthritis from the perspective of people with osteoarthritis | 9-21 |
| **Table S4**. Overview of management of osteoarthritis from the perspective of people with osteoarthritis | 22-29 |
| **Table S5**. Quality assessment with the Critical Appraisal Skills Program (CASP) | 30-32 |
| **Reference list of reviewed studies** | 33-38 |

**Table S1. Search equations**

**Medline via Pubmed**

("Osteoarthritis"[mesh] OR "Osteoarthritis"[tiab] OR "Osteo-arthritis"[tiab] OR osteoarthr*[tiab] OR osteo-arthr*[tiab] OR "osteoarthrosis"[tiab] OR "osteoarthroses"[tiab] OR "degenerative arthritis"[tiab] OR "arthrosis"[tiab] OR "arthroses"[tiab]) AND (qualitative[tiab] OR interview[tiab] OR "white book"[tiab] OR "focus group"[tiab] OR qualitative research[MeSH] OR”perspectives”[tiab] OR “concept mapping”[tiab] OR “online questionnaire”[tiab] OR “online survey”[tiab] OR “online question”[tiab]) AND (pain[MeSH] OR pain[tiab]).

Embase: ('osteoarthritis'/exp OR 'osteoarthritis':ti,ab,kw OR 'osteo-arthritis':ti,ab,kw OR osteoarthr*:ti,ab,kw OR 'osteo arthr*':ti,ab,kw OR 'osteoarthrosis':ti,ab,kw OR ‘osteoarthroses':ti,ab,kw OR 'degenerative arthritis':ti,ab,kw OR 'arthrosis':ti,ab,kw OR 'arthroses':ti,ab,kw) AND ('pain'/exp OR 'pain':ti,ab,kw) AND ('qualitative research'/exp OR 'focus group'/exp OR 'interview'/exp OR qualitative:ti,ab,kw OR interview:ti,ab,kw OR 'white book':ti,ab,kw OR 'focus group':ti,ab,kw OR 'perspectives':ti,ab,kw).

**Cochrane**

#1 AND #2 AND #3 AND #4 AND #5

#1 (osteoarthritis):ti,ab,kw AND (pain):ti,ab,kw AND (qualitative):ti,ab,kw (N=186)

#2 (osteoarthritis):ti,ab,kw AND (pain):ti,ab,kw AND (focus group):ti,ab,kw (N=409)

#3 (osteoarthritis):ti,ab,kw AND (pain):ti,ab,kw AND (perspectives):ti,ab,kw (N=179)

#4 (osteoarthritis):ti,ab,kw AND (pain):ti,ab,kw AND (opinions):ti,ab,kw (N=116)

#5 (osteoarthritis):ti,ab,kw AND (pain):ti,ab,kw AND (views):ti,ab,kw (N=232)

**PsycINFO**

Osteoarthritis AND pain AND qualitative research N=91

Osteoarthritis AND pain AND perspectives or views or perceptions or attitudes or opinion N=405

Osteoarthritis AND pain AND focus group N=43

Osteoarthritis AND pain AND white book N=0

Osteoarthritis AND pain AND interview N=247

**CINAHL**

(MH "Osteoarthritis+" OR AB ("Osteoarthritis" OR "Osteo-arthritis" OR osteoarthr* OR osteo-arthr* OR "osteoarthrosis" OR "osteoarthroses" OR "degenerative arthritis" OR "arthrosis" OR "arthroses") OR TI ("Osteoarthritis" OR "Osteo-arthritis" OR osteoarthr* OR osteo-arthr* OR "osteoarthrosis" OR "osteoarthroses" OR "degenerative arthritis" OR "arthrosis" OR "arthroses") OR SU ("Osteoarthritis" OR "Osteo-arthritis" OR osteoarthr* OR osteo-arthr* OR "osteoarthrosis" OR "osteoarthroses" OR "degenerative arthritis" OR "arthrosis" OR "arthroses"))

AND (MH “pain+” OR TI “pain” OR AB “pain” OR SU “pain”)

AND (MH ("Qualitative Studies+" OR "Interviews+" OR "Focus Groups") OR AB (“qualitative” OR interview OR "white book" OR "focus group" OR "perspectives") OR TI (“qualitative” OR interview OR "white book" OR "focus group" OR "perspectives") OR SU (“qualitative” OR interview OR "white book" OR "focus group" OR "perspectives")).

**Scopus**

(INDEXTERMS(osteoarthr*) OR TITLE-ABS(osteoarthr*) OR TITLE-ABS(osteo-arthr*) OR TITLE-ABS(osteoarthrosis) OR TITLE-ABS(“degenerative arthritis") OR TITLE-ABS (arthrosis)) AND (TITLE-ABS(qualitative) OR TITLE-ABS(interview) OR TITLE-ABS("white book") OR TITLE-ABS("focus group") OR INDEXTERMS(“qualitative research”) OR TITLE-ABS(”perspectives”) OR TITLE-ABS(“concept mapping”)) AND (TITLE-ABS(pain) OR INDEXTERMS(pain)).

**Web of Science**

(AK=osteoarthr* OR TS=osteoarthr* OR TS=osteo-arthr* OR TS=osteoarthros* OR TS=(“degenerative arthritis") OR TS=arthros*) AND (TS=qualitative OR TS=interview OR TS=("white book") OR TS=("focus group") OR AK=(“qualitative research”) OR TS=(”perspectives”) OR TS=(“concept mapping”)) AND ((TS=pain) OR (AK=pain)). Limits: articles and reviews.

**Table S2**. The 79 included studies including the assessment of the quality of the reports with the consolidated criteria for reporting qualitative research (COREQ) checklist

| **Authors** | **Country** | **Qualitative method** | **OA location** | **Number of participants with OA** | **COREQ** |
| --- | --- | --- | --- | --- | --- |
| Agaliotis 2018 [1] | Australia | Focus group. 1 hour session | Knee OA | 11 | 21 |
| Alanazi 2023 [2] | Australia | Semi-structured interviews via zoom | Other | 23 | 25 |
| Baird 2000 [3] | USA | Semi-structured interviews. Interview guide | Other | 18 | 17 |
| Barg-Walkow 2013 [4] | USA | Structured interviews | Other | 8 | 8 |
| Binnie 2022 [5] | Australia | Semi-structured interviews. | Knee OA | 51 | 26 |
| Booker Int J Nurs Stud 2021 [6] | USA | Individual interviews | No precision | 18 | 19 |
| Booker Pain Med 2009 [7] | USA | Individual interviews | No precision | 18 | 17 |
| Booker Geriatr Nurs 2021 [8] | USA | Individual interviews | No precision | 18 | 12 |
| Booker J Fam Nurs [9] | USA | Semi-structured interviews | No precision | 18 | 21 |
| Booker Glob Qual Nurs Res 2020 [10] | USA | Semi-structured interviews | No precision | 18 | 18 |
| Brembo 2016 [11] | Norway | Individual semi-structured interviews | Hip OA | 13 | 19 |
| Buhler 2021 [12] | New Zealand | Semi-structured interviews | Hip OA | 30 | 26 |
| Bukhave 2014 [13] | Denmark | Interviews | Hip OA | 15 | 15 |
| Bunzli 2019 [14] | Australia | Face-to-face and telephone interviews | Knee OA | 27 | 21 |
| Busija 2013 [15] | Australia | Concept mapping: brain storming, sorting, | No precision | 26 | 20 |
| Carmona-Terés 2017 [16] | Spain | Face-to-face individual interviews | Knee OA | 10 | 23 |
| Cedraschi 2013 [17] | France | Focus group | No precision | 14 | 25 |
| Chan 2011 [18] | Hong Kong | Interviews | Knee OA | 20 | 18 |
| Ching 2023 [19] | UK | Semi-structured telephone interviews | No precision | 15 | 29 |
| Darlow 2018 [20] | New Zealand | Semi-structured interviews | Knee OA | 13 | 32 |
| Dharmasri 2020 [21] | USA | Patient intervention feedback open-ended questions | No precision | 93 | 30 |
| Erwin 2018 [22] | UK | Focus group | No precision | 25 | 13 |
| Gay 2018 [23] | France | Semi-structured interviews and focus group | Knee OA | 27 | 32 |
| Gooberman-Hill 2007 [24] | UK | Focus group | Knee OA  Hip OA | 28 | 20 |
| Grime 2010 [25] | UK | Semi-structured interviews | No precision | 27 | 17 |
| Hawker 2008 [26] | Canada | 28 Focus groups and 8 interviews | Knee OA  Hip OA | 143 | 11 |
| Hill 2010 [27] | UK | Semi-structured interviews | Hip OA | 29 | 17 |
| Hinman 2023 [28] | Australia | Semi-structured interviews | Knee OA | 26 | 25 |
| Hiscock 2010 [29] | Australia | Survey | No precision | 38 |  |
| Kanavaki 2022 [30] | UK | Semi-structured interviews | Knee OA  Hip OA | 12 | 32 |
| Kao 2012 [31] | Taiwan | Interviews | No precision | 17 | 18 |
| Kao 2014 [32] | Taiwan | Interviews | No precision | 17 | 22 |
| Kennedy 2022 [33] | Canada | Semi-structured interviews | KneeOA,  Hip OA | 13 | 25 |
| King 2022 [34] | Canada | Semi-structured interviews | Knee OA | 18 | 21 |
| Kline 2012 [35] | USA | Interviews | No precision | 16 | 22 |
| Klinger 1999 [36] | Canada | Structured interviews | Knee OA  Hip OA | 30 | 8 |
| Lawford 2022 [37] | Australia | Semi-structured interviews | Knee OA | 22 | 25 |
| Lenhard 2022 [38] | USA | Focus groups and individual interviews | Knee OA | 15 | 19 |
| Magnussen 2023 [39] | Norway | Interviews | Hip OA | 21 | 25 |
| Maly 2007 [40] | Canada | Interviews | Knee OA | 3 | 24 |
| Manias 2007 [41] | Australia | Focus group | No precision | 34 | 18 |
| McGruer 2019 [42] | Nez Zealand | Semi-structured interviews | Knee OA  Hip OA | 7 | 13 |
| McKevitt 2022 [43] | UK | Semi-structured interviews | No precision | 17 | 22 |
| Miller 2020 [44] | USA | Face-to-face interviews | Knee OA  Hip OA | 11 | 20 |
| Morden 2015 [45] | UK | Interviews | No precision | 22 | 12 |
| Nilsing Strid 2020 [46] | Sweden | Semi-structured telephone interviews | Knee OA  Hip OA | 20 | 27 |
| O'Brien 2023 [47] | Australia | Constructing composite stories | No precision | 25 | 23 |
| Okma-Keulen 2001 [48] | The Netherlands | Semi-structured interviews | No precision | 20 | 27 |
| Olsen 2021 [49] | Norway | Open-ended questions | Hip OA | 35 | 20 |
| Ong 2011 [50] | UK | Semi-structured interviews | No precision | 22 | 12 |
| Panter 2021 [51] | UK | Semi-structured interviews | Hip OA | 30 | 22 |
| Parry 2022 [52] | UK | Semi-structured interviews | Knee OA | 15 | 18 |
| Petursdottir 2010 [53] | Iceland |  | No precision | 12 | 28 |
| Pouli 2014 [54] | UK | Semi-structured interviews | Knee OA | 24 | 20 |
| Power 2008 [55] | Canada | Focus groups | Knee OA  Hip OA | 46 | 17 |
| Puia 2014 [56] | USA | Questionnaire | No precision | 74 | 16 |
| Richardson 2014 [57] | UK | Baseline interviews and monthly contact | No precision | 27 | 15 |
| Romer 2000 [58] | USA |  | No precision | 10 |  |
| Roseman 2006 [59] | Germany | Semi-structured interviews | No precision | 20 | 12 |
| Ryan 2013 [60] | UK | Semi-structured interview. Focus group | No precision | 5 | 21 |
| Sale 2006 [61] | Canada | Interviews | No precision | 19 | 24 |
| Shah 2022 [62] | USA | Focus groups | Knee OA | 35 | 29 |
| Singh 2021 [63] | USA | Focus groups | Knee OA | 48 | 22 |
| Stamm 2009 [64] | Austria, Netherlands, UK, Norway, Sweden | Focus groups | Hip OA | 56 | 20 |
| Stone 2017 [65] | Canada | Semi-structured interviews | No precision | 15 | 21 |
| Swift 2002 [66] | UK | Interviews | No precision | 5 | 18 |
| Sylwander 2022 [67] | Sweden | Semi-structured interviews | Knee OA | 22 | 22 |
| Thomas 2013 [68] | UK | Semi-structured interviews | Other | 11 | 19 |
| Thorstensson 2006 [69] | Sweden | Interviews | Knee OA | 16 | 23 |
| Thumboo 2017 [70] | Singapore | Focus group | Hip OA | 26 | 16 |
| Tollefsrud 2020 [71] | Norway | Focus group | Knee OA  Hip OA | 12 | 19 |
| Uritani 2021 [72] | Japan | Semi-structured interviews | Knee OA | 9 | 24 |
| Wallis 2019 [73] | Australia | Semi-structured interviews | Knee OA | 21 | 27 |
| Woolhead 2010 [74] | UK | Focus group | Knee OA  Hip OA | 123 | 18 |
| Yang 2023 [75] | Singapore | Semi-structured interviews | Knee OA | 46 | 25 |
| Yeowell 2021 [76] | UK | Semi-structured interviews | Other | 9 | 29 |
| Yu 2016 [77] | Dominican Republic | Semi-structured interviews. Interview guide | Knee OA  Hip OA | 20 | 13 |
| Zamanzadeh J Care Sci 2017 [78] | Iran | Interviews | No precision | 19 | 22 |
| Zamanzadeh Drug Res 2017 [79] | Iran | Interviews | No precision | 17 | 20 |

**Table S3**. Overview of features of osteoarthritis from the perspective of people with osteoarthritis

**Domain * Theme * Code *** Quotations

| **Disease** | | |
| --- | --- | --- |
|  | **Chronicity** | |
|  |  | **Incurable**  you can’t change osteoarthritis (Darlow 2018) [20]  It’s incurable, if that’s the word, and you’ve got to live with it and therefore manage it (Hinman 2023) [28]  Every day, consistent in morning (Hawker 2008) [26]  Arthritis cannot be cured (Zamanzadeh Drug Res 2017) [79]  **Progressive**  It wasn’t that bad in the beginning, but pretty soon afterwards, it took a turn for the worse (Brembo 2016) [11]  Wear and tear of the body (Grime 2010) [25]  I don’t want to be crippled (Zamanzadeh J Care Sci 2017) [78]  Progressive: It's worse all the time (Baird 2000) [3]  I’m not old, and these [thumbs]…will get worse as time goes on, cause age will make them worse (Buhler 2021) [12] |
|  | **Life change** | |
|  |  | **Initial processing of having a disease**  It’s just made me realize some things are going to get harder to do (Richardson 2014) [57]  I think, number one, I think it’s the knowledge, no motivation, and number three, just really disbelief. … Disbelief in that, since the father had it, the cousin had it, and everybody in the family had it, now it’s my turn (Booker Int J Nurs Stud 2021) [6]  My thumbs started aching, I thought it was just old age, wear and tear (Grime 2010) [25]  **Not being able to do what one used to do**  It’s an inability to do things I could do in the past (Buhler 2021) [12]  There are so many things I can't do that I used to do. (Baird 2000) [3]  I realized that I couldn’t do it, not that I didn’t want to, I just couldn’t anymore (Stone 2015) [65]  I didn’t realize I’d lost my grip (Buhler 2021) [12]  Oh, I won’t be able to do that, and that…and I’d planned in retirement (Buhler 2021) [12]  My life is somewhat restrictive (Romer 2000) [58]  **Change of lifestyle**  Complete change in lifestyle is required (Roseman 2006) [59]  Decreased ability to maintain a healthy lifestyle (Busija 2013) [15] |
| **Symptoms** | | |
|  | **Pain** | |
|  |  | **Severity**  Level pain is annoying (Alanazi 2023) [2]  I’m paining like hell (Booker J Fam Nurs 2019) [9]  The pain is horrific. It’s just terrible (Yeowell 2021) [76]  It hurts so much I want to cry (Romer 2000) [58]  It’s only minimal pain, not much, but there’s never not pain (Parry 2022) [52]  **Type**  Hurting was like aching and burning, but sometimes sharp and often excruciating (Baird 2000) [3]  The pain is just an aching, dull pain that’s always there (Panter 2021) [51]  It hurts all over (Puia 2014) [56]  I feel just one mass of pain (Pouli 2014) [54]  **Relief**  No effective pain relief (Busija 2013) [15]  Well, if you do nothin’, it’s still gonna hurt (Booker Pain Med 2019) [7]  I do not know if exercise makes it better or worse (Thorstensson 2006) [69]  It just becomes more and more painful (Thorstensson 2006) [69]  **Constant/Intermittent**  That it’s always sitting there in the background (Buhler 2021) [12]  I got [pain] nearly all the time there (Gooberman-Hill 2007) [24]  I had hip pain for a longer period of time (Brembo 2016) [11]  Painful existence (Okma-Keulen 2001) [48]  I don’t know what it is like to be without pain (Pouli 2014) [54]  Usually persistent: I was never free of pain (Baird 2000) [3]  It’s just always there and it just hurts (Yeowell 2021) [76]  I have pain all the time, I just switch off to it (Ching 2023) [19]  I always have pain. That’s part of my life (Sale 2006) [61]  I have more pain at night than I do in the daytime. (Woolhead 2010) [74]  Sometimes I’m fine and others days I’m just oh it really hits... so that’s where it limits me (Shah 2022) [62]  **Unpredictable**  Unpredictable peaks of pain (Cedraschi 2013) [17]  Unsure when pain will come on (Hawker 2008) [26]  **Function-evoked**  If you use your finger or thumb to apply pressure when you do certain things, then the pain usually increases (Thumboo 2017) [70]  I have to be more careful than I used to, my hands are painful all the time when I use them (Stamm 2009) [64]  Pain is a big issue, it stops me being who I want to be and doing the things I want to do (used in 2 categories) (Ryan 2013) [60]  My pain starts as activity starts (Cedraschi 2013) [17]  If I use them the pain gets worse (Romer 2000) [58]  When I move too far to the left, it can be very painful (Olsen 2019) [49]  My knees were very painful when changing from standing to sitting (Morden 2015) [45]  I felt pain when I went upstairs and downstairs (Kao 2012) [31]  It goes when you start moving (Gooberman-Hill 2007) [24]  It just hurts to walk (Sale 2006) [61]  I cannot enjoy walks or exercise because they accentuate my pain (Romer 2000) [58]  Inability to commence a task due to the hesitation about the onset of pain (Agaliotis 2018) [1]  When I go walking, I stop walking when I feel like I can't stand the pain (Uritani 2021) [72]  Pain was both a barrier and motivator (Booker Int J Nurs Stud 2021) [6] |
|  | **Fatigue** | |
|  |  | **General**  You're still fatigued when you wake up, you're still not there (Power 2008) [55]  I’m tired and I can’t keep control my fatigue to do physical activity (Gay 2018) [23]  Feeling tired easily at work (Chan 2011) [18]  And another thing is I have no energy! I haven't been real energetic for a long time (Baird 2000) [3]  I think it's just a complete exhaustion (Power 2008) [55]  **Mental**  The mental fatigue is something much different. You just feel absolutely drained out and you can't focus (Power 2008) [55]  I'm not able...to focus (Power 2008) [55]  It’s hard to get motivated when you are in pain (Singh 2021) [63]  **Pain-evoked**  My pain is exhausting me (Cedraschi 2013) [17]  Knee pain cause fatigue which affected ability to work for a patient with little flexibility in work (Agaliotis 2008) [1]  That level of pain and that level of will power is quite tiring and quite fatiguing (McKevitt 2021) [43] |
|  | **Sleep problems** | |
|  |  | **Disrupted**  Adequate sleep (O’Brien 2023) [47]  Sleep disrupted (Miller 2020) [44]  Affects sleep (Stamm 2009) [64]  I can’t sleep with it, what do I do? (Maly 2007) [40]  **Pain-evoked**  I cannot sleep because of the pain (Cedraschi 2013) [17]  Woken up by pain during sleep (Chan 2011) [18]  When I’m actually in bed, and it will awaken me because I’ll get a sharp pain (Woolhead 2010) [74]  Some nights I felt too much pain. The pain was so severe that I couldn’t sleep. (Zamanzadeh Drug Res 2017) [79]  Sharp pain comes on at night, difficult to sleep (Hawker 2008) [26]  Your sleep pattern is totally disrupted because of pain (Busija 2013) [15]  I can stand a fair bit of pain, but I think I got to the point with it because I was having no sleep, I was just worn out with it in the end (Parry 2022) [52]  **Position-evoked**  You don’t even really sleep much at night because every time you go to turn, it takes an hour to find a comfortable position (Woolhead 2010) [74]  It will wake me up and then I will kind of shake my hand out and move it to another position (Panter 2021) [51] |
|  | **Disability** | |
|  |  | **Damage**  It’s really obvious I have no cushioning in that knee (Darlow 2018) [20]  The knee will seize up a lot more; it’s kind of like a delayed reaction (Woolhead 2010) [74]  I can feel it getting worse. Just doing things, you can feel it clicking and it rubs against one another (Bunzli 2019) [14]  **Frailty**  I wasn’t able to carry it by myself (Bukhave 2014) [13]  I don't trust my knees (Romer 2000) [58]  My knees are powerless. (Kao 2014) [32]  **Getting up/Climbing/Carrying**  Say that people that has severe joint, like my knees are so severe, if I bend down, I cannot get up. (Booker Pain Med 2019) [7]  I’m crippled, I can’t get up (Gooberman-Hill 2007) [24]  When I go to get up, I can't (Baird 2000) [3]  When you are the whanau pani and you can’t get up on your knees, it’s not good (McGruer 2019) [42]  Do not carry heavy stuff (Chan 2011) [18]  **Standing**  When I tried to stand, my knees were so weak I was unable to stand up. Oh, such suffering. (Kao 2012) [31]  Now I do not exercise at all. (Kao 2014) [32]  You can’t walk because you don’t wanna get up. (Booker Glob Qual Nurs Res 2020) [10]  **Pain-evoked disability**  Pain creates physical limitations (Booker J Fam Nurs 2019) [9]  Intense physical pain on a daily basis, and how it negatively affected their desire to be active. (Stone 2015) [65]  Physical fatigue is associated more with, I'd say, aches and pains in their limbs and muscles (Power 2008) [55]  Pain affects ability for physical activity (Busija 2013) [15]  Exercise makes knee pain worse (Singh 2021) [63]  **Activity/speed limitations**  I am limited in my activities (Romer 2000) [58]  numerous adaptations to activities requiring lower extremity mobility (Klinger 1999) [36]  That has led to restrictions on activities (Uritani 2021) [72]  I can manage when doing it at my own speed (Tollefsrud 2019) [71]  It [pain] stops you from doing what you want to do (Stone 2015) [65]  **Walking/mobility**  I noticed on the bad days when I had my aching knee I was slower, I didn't walk as fast (Wallis 2019) [73]  I’m very careful about the way I put my leg, so it doesn’t go out of place (Bunzli 2019) [14]  not being able to walk properly (Gay 2018) [23]  Walking with difficulty (Agaliotis 2018) [1]  To have limited mobility in doing anything (Booker Geriatr Nurs 2021) [8]  Declining ability in walking and standing (Chan 2011) [18]  I can’t walk and it is so painful, it’s horrible (Swift 2002) [66]  I just couldn’t go for a walk (Maly 2007) [40]  **Positioning**  No longer crossed their legs (Klinger 1999) [36]  Certain positions are difficult, and there's probably a way to accommodate it (Miller 2020) [44]  I can no longer sit down on my heels (Uritani 2021) [72]  It has limited me in certain positions (Nilsing Strid 2020) [46]  Can’t keep my legs in one position for a long time (Pouli 2014) [54]  Two participants could no longer wear stockings or socks because they found it too painful or lift their legs (Baird 2000) [3]  **Flexibility**  Little flexibility and that affects the way I walk as well (Alanazi 2023) [2]  Two participants could no longer wear stockings or socks because they found it too painful to lean their legs (Baird 2000) [3]  Stiffness inhibits my movement (Alanazi 2023) [2]  If I sit for too long, when I stand up I’ve really stiffened up. (Kanavaki 2022) [30]  **Balance**  I’ve basically said to him that I can’t do that anymore because I don’ t feel stable enough (Binnie 2022) [5]  I just don’t trust my left at all (Buhler 2021) [12]  Loss of balance, feeling that it won’t support body weight (Hawker 2008) [26]  I can fall over very easily because I’ve got no balance (Alanazi 2023) [2]  **Manual dexterity**  I do like working with my hands and if it’s [finger] really, really painful any particular day, if I’m sitting, I’m moving it, I won’t just let it set, I just move it about (Grime 2010) [25]  Annoying, because sometimes, you know, you can’t hardly grab things (Panter 2021) [51]  The thumb is a big deal in playing (Buhler 2021) [12]  Because I can’t sustain that [wiri] with my hand (Buhler 2021) [12]  Wow – it’s difficult to fix those straps! […] It’s a battle every time. (Bukhave 2014) [13] |
| **Functioning** | | |
|  | **Daily activities** | |
|  |  | **General limitations**  I can no longer do it (Hill 2010) [27]  I still want to do things, but my knees won’t listen (Tollefsrud 2019) [71]  it’s the inability to, that I can’t do things that I find is more debilitating than the actual pain (Parry 2022) [52]  Pain inhibits every single part of your life (Ryan 2013) [60]  is not being able to do stuff (Buhler 2021) [12]  **Restriction of fun things**  Can’t do fun things because of pain (Stamm 2009) [64]  I cannot do things I enjoyed doing (Thumboo 2017) [70]  I can’t run, I can’t ride my bike, I can’t go jogging. So, I can’t enjoy life’ (Alanazi 2023) [2]  Difficult with playing with grandchildren (Chan 2011) [18]  It stops me playing with the grandchildren (Alanazi 2023) [2]  I haven’t went fishing with my friends (Panter 2021) [51]  The span’s limited…which stops you playing the piano (Buhler 2021) [12]  I get no enjoyment out of it [dancing] like I used to (Yeowell 2021) [76]  Giving up a favorite sport (Okma-Keulen 2001) [48]  **Getting dressed**  I can’t even hardly dress myself (Puia 2014) [56]  When it was really bad […] I couldn’t lift my legs to put socks on, shoes and like trousers and that (Kanavaki 2022) [30]  Sat down to remove socks (Klinger 1999) [36]  Then I have to wear long trousers - grab the trouser leg and force the left leg on top of the right one (Brembo 2016) [11]  **Taking a bath/shower**  I can’t even take baths and stuff (Puia 2014) [56]  Showering has become more difficult, because your hands just won’t (Buhler 2021) [12]  I tell you, I don't take baths like I used to. I'm afraid. I'm scared of that tub! (Baird 2000) [3]  When I have taken a shower, I have troubles drying my feet. (Brembo 2016) [11]  **Household chores**  I had to quit because I couldn’t get hold of the bed sheets (Bukhave 2014) [13] impacted daily life, including the ability to engage in daily activities (e.g., housework, gardening, and grocery shopping) (O’Brien 2023) [47]  Difficult with cooking because cannot stand for too long (Chan 2011) [18]  Anything domestic, I don’t do it. I could never do it. (Gooberman-Hill 2007) [24]  Can't even do my laundry because I can't walk to the machines (Baird 2000) [3] It's hard to keep the nice house you'd like to because you can't keep it as clean as you'd like. (Baird 2000) [3]  Do less housework (Chan 2011) [18]  **Manual tasks**  I can’t, like, open up a jar or pick up big objects with it. (Panter 2021) [51]  Difficulty with writing due to pain and reduced movement in fingers (Busija 2013) [15]  Every single day my thumb problem impacts on my daily life: biking, driving, twisting tops off jars/bottles, sewing, pincer action (Buhler 2021) [12]  When fueling I can’t open the petrol cap anymore […] (Bukhave 2014) [13]  I can’t hold the needle any longer. (Stamm 2009) [64]  I’ll drop plates, I’ll drop jars, anything that before I would feel…but now my grip is so weak in my right hand that I drop it (Buhler 2021) [12]  I couldn’t hold a hammer. I couldn’t grip the, uh, the tools. (Panter 2021) [51]  I cannot take the change from the table (Thumboo 2017) [70]  **Shopping**  Had a problem at Aldi [supermarket] with shopping trolley tokens (Richardson 2014) [57]  Reduce frequency of shopping (Chan 2011) [18]  If exchange money is put in the coin tray at check out, then I can’t pick it up. […] It can be really tricky. (Bukhave 2014) [13]  **Outdoor functioning**  Physical activity can also be really painful just in everyday activity, I take huge detours to avoid going up the stairs in the underground. (Gay 2018) [23]  I can’t hold firmly on the handlebars […] and the gears have to be twisted so it is not easy for me at all (to bike). (Bukhave 2014) [13]  **Mobility by car**  it’s difficult for me to just get out of the car. (Kao 2014) [32]  It’s no good me parking quite close to someone (Ong 2011) [50]  I can’t go in cabs by myself (Booker Geriatr Nurs 2021) [8]  You have to haul yourself up on the bus, which you've never had to do before (Erwin 2018) [22]  I don't know how long I can drive for though do (McKevitt 2021) [43]  **Living a sedentary life**  I don’t do much. I sit down. It doesn’t hurt a bit when I’m sitting or lying in bed (Maly 2007) [40]  I’m sat in the house, I can’t do nothing (Yeowell 2021) [76]  By doing something it’s gonna make the knee worse, or you’re going to get pain and you’re trying to avoid the pain so you don’t do anything (Kanavaki 2022) [30]  I can sit for a long time if there is a need for that (Magnussen 2023) [39]  I don't hop out of bed (Baird 2000) [3]  I stay at home every day. I have not wanted to go out for a long time (Kao 2012) [31]  I'm all right as long as I don't move (Baird 2000) [3]  Avoid exercise, avoid the pain (Stone 2015) [65] |
|  | **Work** | |
|  |  | **Obstruction by joint problems**  I worked in the goods department which was very tough for my fingers: you have to handle and to take hold of all the boxes (Bukhave 2014) [13]  I probably just won't be able to do harvest (Ching 2023) [19]  I couldn't work as hard as before (Petursdottir 2010) [53]  Impacted work/employment (O’Brien 2023) [47]  I couldn't have done my Occ Health job 5 days a week… (Ching 2023) [19]  Impact on work productivity; including quitting job, changing job, changing the nature of work, absentees from work, reduced work performances (Thumboo 2017) [70]  AVOIDING JOBS: my knees and job opportunities are limited (Agaliotis 2018) [1]  INSECURITY: felt an insecurity or inability of continuing job with knee pain (Agaliotis 2018) [1]  Physical limitations at work in relation to knee symptoms (Agaliotis 2018) [1]  OA interferes with your paid work (Busija 2013) [15]  I'm really sorry, I can't work today (Ching 2023) [19]  **Obstruction by pain**  I had to stop working because of pain (Romer 2000) [58]  Pain inhibits your jobs (Ryan 2013) [60]  The harder jobs with the cleaning were having a negative impact in that they increase pain (Ching 2023) [19]  Appears to be the longer I work the more the pain level (Wallis 2019) [73]  After working hard, I hurt (Kline 2012) [35]  **Reduction of working hours**  I've had to cut my hours down, I was finding it too much just working full-time. (Ching 2023) [19]  I’ve moved from doing full time work to doing permanent part time because I just couldn’t stand for that length of time on my ankles. So, it restricted me financially (Alanazi 2023) [2]  Work productivity is affected (Thumboo 2017) [70]  **Psychological response**  I couldn’t work, so I was losing . . . my self-respect (Thomas 2013) [68]  I was missing work (Lenhard 2022) [38]  It’s helping me be able to work [interpreted as “it’s helping me to be able to work” (Panter 2021) [51]  **Loss of employment**  So now I cannot work and this has affected our income (Kao 2014) [32]  I had to quit my job (Bukhave 2014) [13]  Give up their job (Okma-Keulen 2001) [48]  Need to quit job (Chan 2011) [18]  I had to retire at 62½ because I couldn’t do my job (King 2022) [34]  I'm not able to...work (Power 2008) [55]  Company is willing to employ me or not. (Yang 2023) [75]  **Need to choose other work**  Looking at a job and knowing that it’s no good starting it because you are not going to be able to finish it . . . a job you could have done so easily just two or three years ago. (Hill 2010) [27]  Moving from running a family business restaurant to an office job that was more sedentary, stating “I had to give it up, I could hardly walk” (Agaliotis 2018) [1]  Has to change work tasks, has to change career plans (Stamm 2009) [64]  Changed the way they conducted themselves at work such as by “not being as interactive” (Agaliotis 2018) [1]  **Need of recovery during and after work**  Increased need for recovery during or at the end of work (Agaliotis 2018 ) [1]  Some days I have taken days off because I’ve been too sore (Buhler 2021) [12]  **Provisions**  Provide workers with various forms of exercise at work such as meditation, Pilates, yoga or swimming (Agaliotis 2018) [1]  Changing the way they do work (Agaliotis 2018) [1]  Recover at home using their own individual approaches such as “lifting legs above waist” or using “compression stockings” (Agaliotis 2018) [1]  Lack of health and safety policies within the workplace to assist workers (Agaliotis 2018) [1] |
|  | **Fear** | |
|  |  | **Movement**  I had been afraid to exercise because of the pain (Lawford 2022) [37]  When I’m walking I feel very insecure, I don’t feel safe, very vulnerable (Parry 2022) [52]  I am careful not to fall (Uritani 2021) [72]  I was definitely even afraid of falling (Shah 2022) [62]  I find new ways to move, but I am worried that I am making things worse for myself (Olsen 2019) [49]  I never know how far I can walk and then pain comes (Pouli 2014) [54]  Behaviors to protect themselves from the hurting: "I walk slowly so if the pain hits, I won't fall" and "I seem to be favoring my hip because I noticed that I walk crooked” (Baird 2000) [3]  I do not know if exercise makes it better or worse (Thorstensson 2006) [69]  And I’m going to fall (Parry 2022) [52]  worried I’m going to fall over (Alanazi 2023) [2]  Walking on a footpath can be dangerous (Alanazi 2023) [2]  I’ve modified everything. If it hurts, I won’t do it (Hawker 2008) [26]  With knee pain I can’t go anywhere (Zamanzadeh J Care Sci 2017) [78]  Sometimes it’s that bad I have to stop and go and sit down (Parry 2022) [52]  I didn’t trust myself on the little humps of grass (Maly 2007) [40]  I’m very careful gardening because of bending (Morden 2015) [45]  That’s part of the reason I’m fallin’ so much. There’s literally nothin’ I can do to stop that. I just have to walk slower (Booker Geriatr Nurs 2021) [8]  Just when you try to walk, boom, it gives up on you and you fall (Bunzli 2019) [14]  **Future deterioration**  It’s scaring me, I don’t know, to look forward to, I don’t know what’s going to happen then (Woolhead 2010) [74]  I am still scared about the future (Romer 2000) [58]  I worry about how bad it might get in the future (Romer 2000) [58]  I kind of consider my future prospects as dark if I have to live like this the rest of my time! (Brembo 2016) [11]  WORRIED, irritated, annoyed by the unpredictability of future progression, unpredictability of future TKR (Agaliotis 2018) [1]  It worries me that one day I won’t be able to do the things I can do today (Darlow 2018) [20]  The fear that my knee may lock or whatever prevents me from doing something (Shah 2022) [62]  I had my mother sitting in a chair for many years as a result of arthrosis. That’s what I fear most (Carmona-Terés 2017) [16]  I did some exercises with trepidation for fear of causing my back pain (Hinman 2023) [28]  Fear of losing ability to do things in future (Hawker 2008) [26]  The worse thing for me is worrying if I’ll end up in a wheelchair (O’Brien 2023) [47] |
| **Psychological** | | |
|  | **Emotional distress** | |
|  |  | **Fear of pain**  Fear of pain during and after physical activity (Stone 2015) [65]  Fear of pain (Nilsing Strid 2020 and Yang 2023) [46, 75]  I anticipate that I was going to suffer you know (Wallis 2019) [73]  I'm frightened to go…I daren't just because of the pain in my back (McKevitt 2021) [43]  **General emotional problems**  Psychological problems, including mental and emotional problems, such as anxiety, fear, frustration, feeling worn out, anger, sadness (Stamm 2009) [64]  Embarrassing because of pain and stiffness while travelling (Yang 2023) [75]  Pain creates emotional distress (Booker J Fam Nurs 2019) [9]  Annoying, irritating, debilitating, stressful, draining, embarrassing, depressing (Hawker 2008) [26]  **Depressive symptoms**  I would be in misery because the pain is so intense. (Kline 2012) [35]  There is always a depressive component. The relation between depression and arthritis pain and physical sensation is an important one. (Roseman 2006) [59]  It makes me sad because it limits me (Carmona-Terés 2017) [16]  Mentally it drags you down (Kanavaki 2022) [30]  No longer very depressed and no longer taking depression meds (Dharmasri 2020) [21]  I tend to get depressed or upset with myself (Power 2008) [55]  I was just getting so depressed with it. (Hill 2010) [27]  I was so depressed! (Yang 2023) [75]  I feel sad, downhearted, depressed (Thumboo 2017) [70]  I got quite depressed with it all. (Yeowell 2021) [76]  My mood is very bad when my leg hurts (Yang 2023) [75]  But, mainly I don't want to get up anymore. (Baird 2000) [3]  Some mornings I wake up and wish I hadn’t woken up, but once I’m out, up and about, that feeling goes. (Richardson 2014) [57]  It gets you down (Hill 2010) [27]  **Anxiety**  When I first start hurting, I get anxious. (Dharmasri 2020) [21]  I only felt scared (Yang 2023) [75]  More anxious about what’s going on, sometimes waking up in sheer anxiety. (O’Brien 2023) [47]  **Worry**  I am not very worried about my knee collapsing on me, but I’m a little worried that the condition of my knee will continue to deteriorate (Binnie 2022) [5]  I see this worries, feelings and thoughts. (Shah 2022) [62]  Worried (Chan 2011) [18]  I am worried, I sometimes think that if it hurts when I do something it will cause even more damage (Thorstensson 2006) [69]  I’m worried that my thumb will be totally deformed (Thumboo 2017) [70]  **Irritation**  Yes, that's horrible. It's a horrible feeling, it's frustrating and probably it makes us more irritable (Erwin 2018) [22]  Prior to the program, the pain would change me. I would go to words and get frustrated. (Dharmasri 2020) [21]  I become irritable, impatient, and I loose temper easily (Thumboo 2017) [70]  Easily get annoyed and argue on minor things with the family / loved ones (Chan 2011) [18]  I was in agony and I still had to unpack the car and wash the gear off (Ong 2011) [50]  I get angry when I can't do something (Romer 2000) [58]  Quite angry with it because you…you use this [thumb] all the time (Buhler 2021) [12]  And that’s the irritating thing…that’s when I start getting grumpy and then I just disappear out the family’s way, because I’m a nasty swine. (Buhler 2021) [12]  That’s how angry it made me, I just walked out of the appointment (O’Brien 2023) [47]  Easily get irritation (Chan 2011) [18]  **Frustration**  I am frustrated when I can't do something (Romer 2000) [58]  I feel frustrated (Thumboo 2017) [70]  It limits me in what I can do … very much so … which is frustrating (Pouli 2014) [54]  Frustration at not being able to do things (Busija 2013) [15]  It’s frustrating not being able to accomplish as much as I’d like to some days (Buhler 2021) [12]  it’s quite depressing cos if I’m planning to do something and then I sort of can’t because it’s started to hurt (Parry 2022) [52]  Just, frustration as much as anything else…it’s bloody annoying at times. (Buhler 2021) [12]  I'm quite frustrated now because of my hip, I can't get up and go (McKevitt 2021) [43]  Definite discontent with having difficulty doing. (Baird 2000) [3]  I was just so desperate to get back to functional. I was missing work…I wanted to run again. I was just feeling very defeated in my daily life, and I wanted to get back to functionality (Lenhard 2022) [38]  I think that’s probably one of the most frustrating things with this is that it actually, it’s not reversible (McGruer 2019) [42]  It’s sheer frustration. (Hill 2010) [27] |
|  | **Self-image** | |
|  |  | **Role change**  I feel a fraud, because I don’t feel as though I’m an arthriticky person. (Richardson 2014) [57]  Pain is a big issue, it stops me being who I want to be and doing the things I want to do (Ryan 2013) [60]  I used to be the custodian and I gave it up (McGruer 2019) [42]  Yeah, you didn’t used to get that grumpy nan (McGruer 2019) [42]  Now I’m the handbrake. (Yeowell 2021) [76]  It’s not just about getting a salary. It’s part of our identity (Tollefsrud 2019) [71]  **Self-esteem**  Sense of shame or embarrassment is colloquially termed “loss of face” (Yang 2023) [75]  You feel a bit diminished as a person (Hill 2010) [27]  I think about them all the time, because when I’m going out, I think ‘do my bunions stick out, do my shoes look alright (Thomas 2013) [68]  I’m troubled by the lack of confidence because of the limiting factors of what I can do with my knee (Binnie 2022) [5]  I am wearing ugly shoes because of arthritis (Romer 2000) [58]  I’m just useless, just because of a daft ankle (Yeowell 2021) [76]  I feel guilty (Thumboo 2017) [70]  **Shame**  I don’t want him to be embarrassed (Kanavaki 2022) [30]  Quite embarrassing because I didn’t like the idea of people seeing me with a walking stick (Yang 2023) [75]  Worries and feels shame (Aboriginal English word used to describe embarrassment) about being seen to not be able to do things (O’Brien 2023) [47]  I don’t want them to think like, there she is, walking around everywhere when she’s too sick to be at work (Tollefsrud 2019) [71]  I was concerned about what others would think (Romer 2000) [58]  I feel embarrassed (Thumboo 2017) [70]  inability to stand and chat at social functions could be interpreted as unfriendliness (Swift 2002) [66]  Well I’m not walking right, and not doing what I, I feel like people are looking (Maly 2007) [40]  **Body image**  Could not afford to wear the jewellery because of the terrible appearance of our hands (Stamm 2009) [64]  I don’t like limping . . . I think it don’t look nice (Thomas 2013) [68]  I don’t want people to know that I’m already so old, half-handicapped (Yang 2023) [75]  I am aware of how horrible they look but I have to sit with them behind (Hill 2010) [27] |
|  | **Devastating consequences (fatalism)** | |
|  |  | **Unable to move**  It’s sore, it is sore. It’s like ‘Ohhh hell, I need to get up’ and I struggle to get up (McGruer 2019) [42]  I'm unable to do anything now (King 2022) [34]  It cripples me, I can’t do much. When the pain is there, there is a loss there actually (Alanazi 2023) [2]  Can’t do it. Can’t do it anymore. (Hawker 2008) [26]  Sometimes I don't feel like going on (Romer 2000) [58]  I don’t have any daily activities because I can’t get around (Alanazi 2023) [2]  **Overwhelming pain**  Wracking pain all over my body at once. It’s devastating (Booker J Fam Nurs 2019) [9]  The biggest thing for me is that [thepain] is gonna get worse and worse (Binnie 2022) [5]  It just stays there and don’t never stop. I’m gonna feel it either way it goes, 24 hours a day (Booker J Fam Nurs 2019) [9]  That’s enough of the pain, cuz my pain, it don’t never stop (Booker Geriatr Nurs 2021) [8]  I’m always in pain and agony, every movement is a chore (Stone 2015) [65]  **A living nightmare**  It's laborious enough just livin (Booker J Fam Nurs 2019) [9]  Living with arthritis . . . it’s a living nightmare (Pouli 2014) [54]  You’re already dealin’ with enough emotionally, mentally when your whole life has been flipped upside down and it’s somethin’ you know is uncureable (Booker J Fam Nurs 2019) [9]  I’m gonna shoot myself you know (Shah 2022) [62]  I feel like crying. It’s horrible. Everyday of my life; it gets a bit upsetting (Yeowell 2021) [76]  **Helpless and hopeless**  There’s nothing I can do about it. (Booker Int J Nurs Stud 2021) [6]  Used to use exercise to cope with depression, but can’t exercise now because of osteoarthritis (Busija 2013) [15]  It just felt like you were so disempowered because you couldn’t do anything about it. (McGruer 2019) [42]  I feel helpless, loss of control (Thumboo 2017) [70]  But in the end, it’s bone on bone, so there’s nothing to do except not move (King 2022) [34]  But I can’t do those either! I’m so confused. I just find it easier to do nothing (Stone 2015) [65] |
| **Social** | | |
|  | **Social life** | |
|  |  | **Lower income**  careful with money—money was now spent only on essentials and holidays forfeited (Hill 2010) [27]  So now I cannot work and this has affected our income. if I don't go into work, I don't get paid (Kao 2014) [32]  So, it restricted me financially (Alanazi 2023) [2]  Need to use home help, which is extra financial burden (Busija 2013) [15]  **Socializing**  It’s had a terrific effect on the social side of things (Thomas 2013) [68]  Impacted family and social life (O’Brien 2023) [47]  My mood is affecting the relationship with relatives and friends (Thumboo 2017) [70]  I miss the interaction with people (Romer 2000) [58]  **Isolation**  My pain makes me feel isolated and old (Cedraschi 2013) [17]  Now when I don’t row it’s different. One of them, I don’t see anymore and the other one I see very seldom (Bukhave 2014) [13]  we don’t have many guests for dinner anymore (Bukhave 2014) [13]  Avoiding social contact (Stamm 2009) [64]  Our family members shunning me, so you had to deal with the emotional stuff too (Booker J Fam Nurs 2019) [9]  **Communication**  Pain inhibits all the communication you have with people, your relationships (Ryan 2013) [60]  “not being as interactive” [RN] because of pain associated limitation in the knee joint(s) (Agaliotis 2018) [1]  Nobody else wants to hear about it (Power 2008) [55]  I’m in pain all the time, but I don’t let them know. (Booker Glob Qual Nurs Res 2020) [10]  I think that communication between people in the same situation is effective (Uritani 2021) [72]  **Participation**  When my friends say they want to go for a walk, I’ll try not to go  I am unable to take part in social activities I want to (Thumboo 2017) [70]  Prefer staying at home / going out with friends less frequent (Chan 2011) [18]  It’s not easy being at work and not contributing as much as the others. I had this anxiety in me all the time, it was exhausting. (Tollefsrud 2019) [71]  **Change of relationships**  Relation to others, family, neighbours, friends, help from peers (Stamm 2009) [64]  Relationships of friends that maybe has suffered the most (Power 2008) [55]  Personal relationships are affected (Busija 2013) [15]  Could not sleep at night because of her OA pain (Sale 2006) [61]  **Intimacy**  Pain limits sexual health (Nilsing Strid 2020) [46]  Sexual activities must then be adapted, which can limit sexual health (Nilsing Strid 2020) [46]  Fear of pain and avoidance of sexual activities (Nilsing Strid 2020) [46]  Pain creates relationship disruption (Booker J Fam Nurs 2019) [9]  Now that we don’t really have any sex life (Nilsing Strid 2020) [46] |
|  | **Dependency** | |
|  |  | **Need of assistance**  Only possible when assisted by others (Okma-Keulen 2001) [48]  I depend on my husband a lot more now (Hawker 2008) [26]  I’ve had to hang onto my husband many a time (Morden 2015) [45]  I always had to have help (Maly 2007) [40]  Either I keep trying […] or I wait until someone comes by to visit […] or I go out in the hallway and ask (Bukhave 2014) [13]  Need to be accompanied when going out (Chan 2011) [18]  If I was living alone, I would say – I wouldn’t know what the hell to do? Either I would have to live in a nursing home or I would say – have to get an au-pair in the house (Bukhave 2014) [13]  The pain makes me depend on others for help (Romer 2000) [58]  I have to depend on my husband to be able to do something (Yu 2016) [77]  I haven’t got that independence that I had before (Hill 2010) [27]  **Loss of independence**  Just being able to do things on your own without asking for help. Uh, huh, I’d like to be a whole person (Maly 2007) [40]  This loss of independence and freedom of movement is hard to bear (Okma-Keulen 2001) [48] |
|  | **Lack of understanding** | |
|  |  | **Society and work**  I guess men are not all equally understanding (Petursdottir 2010) [53]  You will bury all of us! You are not really ill, you look fine! (Cedraschi 2013) [17]  They really need to understand the pain and how severe it is (Erwin 2018) [22]  I know they mean well, calling me stubborn and such things (Tollefsrud 2019) [71]  RELUCTANCE TO DISCLOSE: risk of being stigmatized because of the disease’s “invisibility” (Agaliotis 2018) [1]  "It's downright pitiful,": negative emotions (such as frustration, anger, and sadness) in their voices and gestures (Baird 2000) [3]  People do not understand them (Chan 2011) [18]  I feel not being understood, other people do not understand what I am suffering  only I know my own pain, other people won’t understand (Thumboo 2017) [70]  Help is good, but I don’t want pity (Booker J Fam Nurs 2019) [9]  The participants did not state "I can't" out of unwillingness but out of impossibility (Baird 2000) [3]  Was left feeling upset and disgusted with herself, What’s worried me most of all was that people thought I was not telling the truth. (Swift 2002) [66]  LACK OF UNDERSTANDING: lack of understanding, empathy, and support among supervisors (Agaliotis 2018) [1]  **Health professionals**  Health professionals need to understand that it is very wearing and tiring, and that it changes your life (Erwin 2018) [22]  She come she said, ‘it's just arthritis I'm afraid it's just something at your age’ (McKevitt 2021) [43]  **Family**  Osteoarthritic pain is real although families can’t physically see or choose not to ‘see’ the pain (Booker J Fam Nurs 2019) [9]  My daughter has adopted a very concerned attitude about my welfare (Grime 2010) [25]  **Partner**  Husband helps versus gets up earlier than the husband to do all the housework (Stamm 2009) [64]  My husband thinks that I am complaining all the time (Romer 2000) [58] |

**Table S4**. Overview of management of osteoarthritis from the perspective of people with osteoarthritis

**Domain * Theme * Code *** Quotations

| **Symptoms** | | |
| --- | --- | --- |
|  | **Cognitive-behavioral** | |
|  |  | **Behavioral pain management**  I'll put under my knees and then I'll put a cold pack on them (Kennedy 2022) [33]  I can generally control what’s bringing the pain on (Binnie 2022) [5]  However, every time I stretched, more tension was released. Now it is less painful (Olsen 2019) [49]  Compensate using the better knee (Agaliotis 2018) [1]  **Distraction**  I try not to even focus on it, so I certainly don’t keep track of it (Barg-Walkow 2013) [4]  If you're in cheerful company and there's something to laugh about, you can forget your fatigue and you can forget your pains (Power 2008) [55]  **Sleep**  I might jump in the bath in the middle of the night…and then I’ll be able to sleep another couple of hours (McGruer 2019) [42]  Sometimes sleeping with a hot water bottle, using a knee brace during the day, and using emu oil (O’Brien 2023) [47]  I sleep with the pillow between, but if it slips away or something and there’s pressure on the knee, that will, it will hurt enough to wake me (Woolhead 2010) [74] |
|  | **Pharmacological and surgical** | |
|  |  | **Use of medication**  I always took analgesic medicine (Kao 2012) [31]  I suppose I’d come home and take half a pill or full pill, and just stay home. (Kline 2012) [35]  Okay, I am dependent, for my pain I am depend on medication, without medication I would be really limited, maybe 60% (Maly 2007) [40]  I used to take [painkillers] more often (Sale 2006) [61]  That’s when I usually run in and get injections, because it just hurts so bad (Lenhard 2022) [38]  I used to be on tramadol and codeine and all of that (Buhler 2021) [12]  **Medication as a last resort**  Sometimes I can’t control it, and then I go to the analgesics (Yu 2016) [77]  I have some, in case one day it hurts too much (Carmona-Terés 2017) [16]  When I take codeine, I’m much better for 24 h and don’t feel any pain, but this drug has side-effects and I don’t take it permanently even when I have severe pain. (Zamanzadeh Drug Res 2017) [79]  Quite a lot of my pain medications are taken just when necessary (Manias 2007) [41]  On a bad day, I have to stop and find something to take [not sure whether this is analgetic medication] (Booker Pain Med 2019) [7]  I have to take painkillers but I am not a great advocate (Pouli 2014) [54]  Only take a pill when I am in terrible pain, otherwise I am against drugs. (Roseman 2006) [59]  **Always pain**  Medicine helps some, but not much, I am constantly in pain (Singh 2021) [63]  I mean, you have the painkillers and it improves it but it never goes away (Pouli 2014) [54]  **Fear and avoidance of medication**  I avoid taking pills (Carmona-Terés 2017) [16]  I don’t take things like that (Percodan); That is a hard drug (Sale 2006) [61]  I’m also very afraid of taking medications (Miller 2020) [44]  I’m like I’d rather live with that disease than all the side effects (Lenhard 2022) [38]  I believe that if you take painkillers you’ll become worse and get more pain in the end (Brembo 2016) [11]  With painkillers, you won’t feel the pain and then the situation gets worse - I believe so anyway....... I would rather choose to rest – when you have had some rest, the pain isn’t so bad (Brembo 2016) [11]  It’s not ok to carry on like that....... But they....... They just prescribe pills- and I’m not a pill person.......(Brembo 2016) [11]  You know, when you have taken pills over a period of time you’ll eventually need even stronger ones (Brembo 2016) [11]  I try to stick to that because I don’t wanna have too much I’m on heart pills and other pills as well. (Buhler 2021) [12]  They use each medication expecting a miracle (Zamanzadeh J Care Sci 2017) [78]  **Cautious use of medication**  I try not to take medication every day (Yu 2016) [77]  I don’t want to get addicted either. (Sale 2006) [61]  But I am careful; if I can take the pain then I won't take a pill because they are not really good for you. (Roseman 2006) [59]  But I don’t take a Tylenol then. I just walk to the kitchen. (Sale 2006) [61]  I’ve got a lot of drugs to take and when I am getting close to the next lot I get anxious (McGruer 2019) [42]  **Sleep medication**  Because I find I take painkillers more to sedate myself in the hopes of getting to sleep rather than, because it doesn’t really take away the pain (Woolhead 2010) [74]  I have to be competent, I have to be alert, I have to think, so I absolutely refuse to take anything in the daytime. But I take it at night because if I didn’t, my body would never relax for me to actually fall asleep (Woolhead 2010) [74]  **Surgery as a last straw**  I’m worried about the money, that’s what took me so long to get my shoulder done (Binnie 2022) [5]  Really don’t want to have an arthrodesis, and that’s expensive (Alanazi 2023) [2]  So, it bothers me a lot....... that is why I went to the doctor and said that we need to fix my hip. I can’t live with this – its impossible (Brembo 2016) [11]  When you have suffered long enough you’ll try almost anything…[it is about wanting surgery] (Brembo 2016) [11]  Knees were a small thing not to worry about or a funny joint that can be surgically replaced compared to ankle or back (Agaliotis 2018) [1]  **Miscellaneous**  I have been thinking should I be taking Celebrex, as one doctor said it was okay to keep taking it, and then I saw another doctor and he said to stop taking it. (Manias 2007) [41]  Adherence to pills: not consistent, not taking the meds the same time every day (Singh 2021) [63]  it can only be treated with calcium and vitamins (Zamanzadeh Drug Res 2017) [79] |
| **Functioning** | | |
|  | **Physical activity/exercise** | |
|  |  | **Walking/movement**  The only thing that would stop me from walking is the pain barrier. (Wallis 2019) [73]  The more you move, the better it feels (Lawford 2022) [37]  I know that simply going for a walk every day is very good for me (Thorstensson 2006) [69]  If I move a lot, it gets better. (Kline 2012) [35]  When I have been walking for a while, the pain goes away (Thorstensson 2006) [69]  I have to choose not to run, so I walk instead, and it’s better than nothing. (Sylwander 2022) [67]  It’s keeping you in motion, keeping you active, and it’s not stress (Darlow 2018) [20]  If you want to keep your mobility you’ve got to move, even if you’re in pain, you’ve got to move. (Richardson 2014) [57]  Now we are putting everything together so that the whole body is moving, and it is like something is released in the body. (Olsen 2019) [49]  We made more of an effort to get out for a walk, and back home (Richardson 2014) [57]  Sports are helpful, such as Nordic walking (Stamm 2009) [64]  Keep myself moving (Shah 2022) [62]  I think it’s just lying in bed that sort of stiffens the leg up, and as I say, seconds or so after I’m up and moving it’s alright then (Woolhead 2010) [74]  **Exercise**  Exercising to manage the knee pain (Sylwander 2022) [67]  Finding exercise helpful for pain relief (Booker Int J Nurs Stud 2021) [6]  Only be improved by doing strengthening exercises (Hinman 2023) [28]  I take care of myself with light exercises to prevent its progress and reduce pain  have to do exercise every morning (Zamanzadeh J Care Sci 2017) [78]  The muscles are shaking, but it feels good to sense them working again. (Olsen 2019) [49]  These are healthy movements, kind to my hip (Olsen 2019) [49]  It felt nice. I was focusing on the slow movements and did not force anything. (Olsen 2019) [49]  I know exercise helps me. (Booker Geriatr Nurs 2021) [8]  Make my knees work, it would help and it seems to do (Kanavaki 2022) [30]  Like to exercise, but must choose the mode of exercise (Kao 2014) [32]  **Exercise in water**  Water exercise: It’s very beneficial because I really can move without hurting myself as opposed to doing land aerobics. (Booker Int J Nurs Stud 2021) [6]  Swimming reduced my pain (Zamanzadeh J Care Sci 2017) [78]  Swimming was also good. (Kao 2014) [32]  **Challenging physical limits**  people ask me why I am huffing and puffing. I don’t stop until I have finished whatever I am doing, but afterwards; I am totally worn out (Olsen 2019) [49]  Just keep on living, and I say “You just need to hush” (Booker J Fam Nurs 2019) [9]  I can’t slow down, and take my time. (Buhler 2021) [12] |
|  | **Assistive devices** | |
|  |  | **Rollator/cane/brace**  I know I can balance well [with a walker] because I am worried about doing more harm if I fell (Bunzli 2019) [14]  If I’m in the kitchen or whatever, I’ll sit. I have a walker with wheels on it and a seat on it. (Booker J Fam Nurs 2019) [9]  Assistive devices to walk out doors: I use a cane all the time (Baird 2000) [3]  I must use [knee] braces to stand back up (Kao 2014) [32]  There are ways to be mobile. (Kanavaki 2022) [30]  **Manual aids**  My husband used to cut bread but when we got the slicer then suddenly I was able to cut all kinds of bread and vegetables (Bukhave 2014) [13]  I have had to put a strap to the accelerator handle so it is jammed when I mow the lawn because I can’t keep it pressed during mowing (Bukhave 2014) [13]  I simply can’t live without it. I always bring my knife in a paper tissue - because then I can take part (Bukhave 2014) [13]  **Adaptation of furniture**  it’s quite deliberate that handles of the cupboard doors have been replaced with thick wooden handles providing enough space for all of my hand and also that they are not cold to hold (Bukhave 2014) [13]  Modified car transfers by avoiding cars with low seats (Klinger 1999) [36] |
|  | **Behavioral adaptation** | |
|  |  | **Coping**  Own personal strategies to help cope with knee pain while working (Agaliotis 2018) [1]  I just try to take control of it myself. Then with assistance from my family (Booker J Fam Nurs 2019) [9]  Don’t get me wrong, I’m not a martyr but I do try [to cope] (Richardson 2014) [57]  I sit on the stool and do a portion (Baird 2000) [3]  Just because I’m old, that don’t mean I can’t change the way I do certain things (Booker Int J Nurs Stud 2021) [6]  We simply had to try different things and find out what worked. (Nilsing Strid 2020) [46]  I can choose to avoid activities posing problems - and then they (problems) don’t appear (Bukhave 2014) [13]  Being active to achieve not only physical but psychological well-being (Gay 2018) [23]  It takes me a little longer to do things (Baird 2000) [3]  I’ll throw some ice on my knees, take a hot bath after. It feels great (Stone 2015) [65]  To reduce knee pain, the best method was to shower in hot water and rest in a hot spring. just deal with it (Kao 2014) [32]  Cleaning the house now requires planning so that less effort will be required (Baird 2000) [3]  I had to change to printing now because of my stiff and hurting fingers (Baird 2000) [3]  **Learned control**  I done dealt with it so long that I’ve adjusted myself to the pain. (Booker Glob Qual Nurs Res 2020) [10]  At first, it was difficult for me to trust my toes, but then I felt quite comfortable and stable (Olsen 2019) [49]  I actually have managed to live with the pain (Brembo 2016) [11]  When I exhale properly, the shoulder tension dissolves. It feels wonderful (Olsen 2019) [49]  **Keeping oneself tough**  I work with people suffering from cancer and I can’t let them see me looking neglected (Gay 2018) [23]  we can be in pain, but we know we got to do the job so we can take care of our family (Booker Glob Qual Nurs Res 2020) [10]  I don't want to show that I can't do the job (Ching 2023) [19]  When you are in pain, you endure it (Yu 2016) [77]  There has to be a balance, I think, and that’s one thing I’ve never got right, that balance of taking a bit of rest when I probably needed rest (Kanavaki 2022) [30]  Don’t give up, keep going. Don’t give up. Don’t let it get on top of you (Richardson 2014) [57]  Then hurting the next day because you tried so hard to impress someone (Shah 2022) [62]  Our grandparents had osteoarthritis. They never complained (Cedraschi 2013) [17]  He wasn’t going to let the pain rule his life (O’Brien 2023) [47]  At least I be in pain, I still get out there and cut my yard! I still get out there in my garden (Booker J Fam Nurs 2019) [9]  Keep on going (Hiscock 2010) [29]  Keeping going in body, mind and everyday life (Richardson 2014) [57]  **Relaxation**  I sit for a while, I relax, and that’s it (Yu 2016) [77]  That was best for me because that way I relaxed my leg (Nilsing Strid 2020) [46]  **Asking help from others** My daughter helped me sell betel nuts and I helped look after her child (Kao 2014) [32]  And you learn more from other people (King 2022) [34]  I called my daughter to touch my knee when my knee was in pain (Kao 2012) [31]  **Planning recovery periods**  I try to limit the number of strenuous activities per week, and spread them out over the days of the week so I don’t overdo it any one day and get the job done without aggravating my osteoarthritis (Barg-Walkow 2013) [4]  If I have a long day one day, I take it a bit easy the next but no one's spoken to me about it. (Erwin 2018) [22]  Dividing jobs up instead of trying to do everything all at once, if you can do only one load of laundry today, fine. (Power 2008) [55]  I need to take a break now because my legs are hurting (Agaliotis 2018) [1]  Doing less / stop / change to different exercise (Chan 2011) [18]  It’s the balance between activity and rest for the joint (Darlow 2018) [20] |
| **Psychological** | | |
|  | **Adjustment** | |
|  |  | **Time will heal**  Well, I can’t do this right now. I have to wait (Booker J Fam Nurs 2019) [9]  I trust in God and the doctor that it will be a success (Yu 2016) [77]  if I’m suffering, then I feel He can heal me, whatever goin’ on (Booker Glob Qual Nurs Res 2020) [10]  Expectations from treatment (Chan 2011) [18]  **Self-management intentions**  It’s impacting on how I would normally live my life, I have to be more aware of what I’m going to do. (Buhler 2021) [12]  I have to overcome [the pain] (Yu 2016) [77]  You’ve got to be active haven’t you, you’ve got to do something. (Parry 2022) [52]  Now you have to plan and make choices (Binnie 2022) [5]  I had made an agreement with myself to try knitting again but with thick knitting needles. (Bukhave 2014) [13]  **Realistic expectations**  I didn’t expect great improvements. (Hinman 2023) [28]  But you’ve got to adjust your life to what you can do (Swift 2002) [66]  **Acceptance**  I must live with that (Magnussen 2023) [39]  If you accept it your attitude changes (Carmona-Terés 2017) [16]  African Americans are probably more capable or better able to accept and deal with pain than others (Booker Glob Qual Nurs Res 2020) [10]  Take it easy old girl, be thankful someone will do it for you and help you. (Swift 2002) [66]  Oh look, I’ve had it for so long, I just, it’s just part of life (Hinman 2023) [28]  **Lifecycle acceptance**  Chronic knee pain as part of old age or being older (Agaliotis 2018) [1] It's part of the ageing process (Kennedy 2022) [33]  It is tied up with me getting older (Buhler 2021) [12]  I think there is an age effect (Uritani 2021) [72]  Wear and tear, you know, you’re getting older (Darlow 2018) [20]  Just, [pain]’s part of old age. Get used to it, and don’t whine about it (Barg-Walkow 2013) [4]  I think that as you get older, you expect such things to occur… things you might have to accept (Brembo 2016) [11]  Symptoms are in relation to age (Stamm 2009) [64]  The will is still there, but you’re ageing. (Gay 2018) [23]  I have long been aware that I would need to replace the hip at some point, but I wanted to wait as long as possible, it’s something that you kind of need to expect when you are so lucky to get old (Brembo 2016) [11]  Arthritis is something you get when you're old (Romer 2000) [58]  I am older now so arthritis is expected (Romer 2000) [58]  I think it’s just because I am getting older (Pouli 2014) [54]  It’s part of aging (King 2022) [34]  **Cognitive restructuring**  Understanding the limitations due to pain (Agaliotis 2018) [1]  It is always there, but it isn’t always severe (Ong 2011) [50]  I do get better, when I forget about the pain (Yu 2016) [77]  My focus should be on other things, and I also try to stay positive! (Brembo 2016) [11]  All recoveries are painful. They are never easy. It all depends on your mentality (Yu 2016) [77]  It’s got to be self. To me, I think a lot of it’s got to be self. If you wanna manage that pain, it’s got to be up here [mind]. I can do this. I’m gonna manage this. (Booker Int J Nurs Stud 2021) [6]  I anticipated that I would expect to get more discomfort (Ong 2011) [50]  There’s always some pain to have a gain (Hinman 2023) [28]  So long as there’s no pain I don’t care what it looks like (Buhler 2021) [12]  Well, I’ve noticed, you know if compared to this one there’s a lot more knobbly bits. But it doesn’t bother me, no (Buhler 2021) [12]  I walked in and I saw all these poor people totally crippled . . . well so many, struggling so much . . . well . . . I walked in and I felt a fraud just being there. You realise how . . . what I’ve got is nothing (Hill 2010) [27]  There are so many worse things than I have (Maly 2007) [40]  **Positive mindset**  Keep a positive mind, live on that day (Swift 2002) [66]  Even though my hands hurt, I am not ill (Magnussen 2023) [39]  Sometimes if you have a positive outlook, things get better (Yu 2016) [77]  It don’t really have much to do with arthritis. I just cope and enjoy the company and the food and the peoples (Booker Geriatr Nurs 2021) [8]  **Self-reliance**  I was fairly confident that would give at least some benefit (Hinman 2023) [28]  I know that when I'm done I feel better (Petursdottir 2010) [53]  That’s what it was. I don’t wanna depend on that (Lenhard 2022) [38] |
|  | **Barriers** | |
|  |  | **Unhelpful thinking**  When I start thinking to myself I’m fed up, I’ve got to stop that and think positive (Richardson 2014) [57]  I can cope during the day because I’ve got things to do, I’m moving all the time, but it’s at night, all of a sudden all you have to think about is how bad your knee hurts (Woolhead 2010) [74]  **Obesity**  Paralyzing, terrorizing, want to scream, want to cry, you get mad about the pain  Person’s self-responsibility regarding “controlling” knee pain [WG] by “keeping weight down” [RN], recognizing the consequence of carrying “extra weight” [JG] to the knee joint as “taking a toll on the body” (Agaliotis 2018) [1] |
|  | **Disappointments** | |
|  |  | **Inability to perform exercise**  I couldn't hands on treat (Ching 2023) [19]  Pain; I had trouble with therapy and exercise with the pain (Singh 2021) [63]  Exercise makes knee pain worse (Singh 2021) [63]  **Treatment**  There is nothing that can be done about the OA; therefore, I do nothing (Petursdottir 2010) [53]  Do not expect that OA treatments will significantly help my symptoms (King 2022) [34]  **Health care professionals**  There’s nobody that appears to be an expert in osteoarthritis (Ryan 2013) [60]  Instructors probably can’t teach me nothing… I would teach them (Booker Int J Nurs Stud 2021) [6]  I was very, very disappointed with the GP (Ching 2023) [19]  I don’t think [my family doctor] knew what to do (King 2022) [34]  I decided to replace this GP immediately, because I thought… I was a bit mad at him - I had lived and struggled with this for so many years without anything being done. He listens to what you say, but haven’t taken it seriously enough (Brembo 2016)  I said I’ve got a lot of pain, I can’t sleep with it, what do I do? Learn to live with it. I love my doctor, he’s wonderful, but that I didn’t like.[I think that these separate statements refer to each other.] (Maly 2007) [40]  GP aren't specialists of OA, I ask about, but I don’t really expect to receive any particular kind of information (Brembo 2016) [11]  I was disappointed that he just touched it and I can’t see how he can tell it’s what it is just by doing that to it (Thomas 2013) [68]  **Communication**  I asked my questions and they told me that they didn’t want to spend time on them (Manias 2007) [41]  He was more interested in taking pictures of my knee than in examining it (Roseman 2006) [59]  Could have made stronger demands in consultations (Magnussen 2023) [39] |
| **Social** | | |
|  | **Social support** | |
|  |  | **General**  I’m alright if my husband’s with me and I can hold his arm (Parry 2022) [52]  My wife is the gogetter here. (King 2022) [34]  Help & support from family members (Chan 2011) [18]  Very helpful and understanding husband (Romer 2000) [58]  Quite nice to accompany me (Yang 2023) [75]  The opportunity to exercise with people with the same complaint (McKevitt 2021) [43]  I know [my husband] supports being active (Stone 2015) [65]  **Instrumental**  I feel less guilty asking for help (Dharmasri 2020) [21]  Letting my family help me even though I am not happy (Hiscock 2010) [29]  The person who helped me was very good in many ways. (Sylwander 2022) [67]  They brought me a chair, they brought me my plastic card, the things to sign, I didn’t have to do a thing, they were lovely. (Swift 2002) [66]  Getting help from my family is not dependence but reality (Romer 2000) [58]  A lot of times I have to have help (Puia 2014) [56]  hire people, help & support from family members (Chan 2011) [18]  **Emotional**  It was good for me that someone said, “I see that it is painful.” I felt I got help, really (Magnussen 2023) [39]  It’s very relaxing to be in the atmosphere of the people and warmth and friendliness (Kline 2012) [35]  They [family] always think of you first, they always want [you] to get better (Yu 2016) [77]  Lots of bonding, lots of support, lots of strength from them, especially from my parents (Yu 2016) [77]  I’m largely by myself, okay, so there’s really nobody for me to go to but Him (Booker J Fam Nurs 2019) [9] |
| **Quotations that were not categorized (difficult to interpret)** | | |
|  |  | When you have OA, your joints already hurt, so I assume they’re not up to it (Gay 2018) [23]  it'd probably do more harm than good (McKevitt 2022) [43]  I regret saying something people don't like to hear (Uritani 2021) [72]  I was talkin’ to this brother that is right over here. (Booker Glob Qual Nurs Res 2020) [10]  You buy things in place of it . . . you look for ways round things. (Hill 2010) [27] I really think it was because I wasn’t doing what I needed to do. (Hinman 2023) [28]  Aware of the adjustments (Richardson 2014) [57]  Because it gives me pain when I do things. That’s the only signal (Binnie 2022) [5]  Need to come up with strategies (Agaliotis 2018) [1] |

**Table S5**. Quality assessment with the Critical Appraisal Skills Program (CASP)

| **Authors** | **Question Numbers (CASP)** | | | | | | | | | |
| --- | --- | --- | --- | --- | --- | --- | --- | --- | --- | --- |
|  | **Q1** | **Q2** | **Q3** | **Q4** | **Q5** | **Q6** | **Q7** | **Q8** | **Q9** | **Q10** |
| Agaliotis 2018 [1] |  |  |  |  |  |  |  |  |  |  |
| Alanazi 2023 [2] |  |  |  |  |  |  |  |  |  |  |
| Baird 2000 [3] |  |  |  |  |  |  |  |  |  |  |
| Barg-Walkow 2013 [4] |  |  |  |  |  |  |  |  |  |  |
| Binnie 2022 [5] |  |  |  |  |  |  |  |  |  |  |
| Booker Int J Nurs Stud 2021 [6] |  |  |  |  |  |  |  |  |  |  |
| Booker Pain Med 2009 [7] |  |  |  |  |  |  |  |  |  |  |
| Booker Geriatr Nurs 2021 [8] |  |  |  |  |  |  |  |  |  |  |
| Booker J Fam Nurs [9] |  |  |  |  |  |  |  |  |  |  |
| Booker Glob Qual Nurs Res 2020 [10] |  |  |  |  |  |  |  |  |  |  |
| Brembo 2016 [11] |  |  |  |  |  |  |  |  |  |  |
| Buhler 2021 [12] |  |  |  |  |  |  |  |  |  |  |
| Bukhave 2014 [13] |  |  |  |  |  |  |  |  |  |  |
| Bunzli 2019 [14] |  |  |  |  |  |  |  |  |  |  |
| Busija 2013 [15] |  |  |  |  |  |  |  |  |  |  |
| Carmona-Terés 2017 [16] |  |  |  |  |  |  |  |  |  |  |
| Cedraschi 2013 [17] |  |  |  |  |  |  |  |  |  |  |
| Chan 2011 [18] |  |  |  |  |  |  |  |  |  |  |
| Ching 2023 [19] |  |  |  |  |  |  |  |  |  |  |
| Darlow 2018 [20] |  |  |  |  |  |  |  |  |  |  |
| Dharmasri 2020 [21] |  |  |  |  |  |  |  |  |  |  |
| Erwin 2018 [22] |  |  |  |  |  |  |  |  |  |  |
| Gay 2018 [23] |  |  |  |  |  |  |  |  |  |  |
| Gooberman-Hill 2007 [24] |  |  |  |  |  |  |  |  |  |  |
| Grime 2010 [25] |  |  |  |  |  |  |  |  |  |  |
| Hawker 2008 [26] |  |  |  |  |  |  |  |  |  |  |
| Hill 2010 [27] |  |  |  |  |  |  |  |  |  |  |
| Hinman 2023 [28] |  |  |  |  |  |  |  |  |  |  |
| Hiscock 2010 [29] |  |  |  |  |  |  |  |  |  |  |
| Kanavaki 2022 [30] |  |  |  |  |  |  |  |  |  |  |
| Kao 2012 [31] |  |  |  |  |  |  |  |  |  |  |
| Kao 2014 [32] |  |  |  |  |  |  |  |  |  |  |
| Kennedy 2022 [33] |  |  |  |  |  |  |  |  |  |  |
| King 2022 [34] |  |  |  |  |  |  |  |  |  |  |
| Kline 2012 [35] |  |  |  |  |  |  |  |  |  |  |
| Klinger 1999 [36] |  |  |  |  |  |  |  |  |  |  |
| Lawford 2022 [37] |  |  |  |  |  |  |  |  |  |  |
| Lenhard 2022 [38] |  |  |  |  |  |  |  |  |  |  |
| Magnussen 2023 [39] |  |  |  |  |  |  |  |  |  |  |
| Maly 2007 [40] |  |  |  |  |  |  |  |  |  |  |
| Manias 2007 [41] |  |  |  |  |  |  |  |  |  |  |
| McGruer 2019 [42] |  |  |  |  |  |  |  |  |  |  |
| McKevitt 2022 [43] |  |  |  |  |  |  |  |  |  |  |
| Miller 2020 [44] |  |  |  |  |  |  |  |  |  |  |
| Morden 2015 [45] |  |  |  |  |  |  |  |  |  |  |
| Nilsing Strid 2020 [46] |  |  |  |  |  |  |  |  |  |  |
| O'Brien 2023 [47] |  |  |  |  |  |  |  |  |  |  |
| Okma-Keulen 2001 [48] |  |  |  |  |  |  |  |  |  |  |
| Olsen 2021 [49] |  |  |  |  |  |  |  |  |  |  |
| Ong 2011 [50] |  |  |  |  |  |  |  |  |  |  |
| Panter 2021 [51] |  |  |  |  |  |  |  |  |  |  |
| Parry 2022 [52] |  |  |  |  |  |  |  |  |  |  |
| Petursdottir 2010 [53] |  |  |  |  |  |  |  |  |  |  |
| Pouli 2014 [54] |  |  |  |  |  |  |  |  |  |  |
| Power 2008 [55] |  |  |  |  |  |  |  |  |  |  |
| Puia 2014 [56] |  |  |  |  |  |  |  |  |  |  |
| Richardson 2014 [57] |  |  |  |  |  |  |  |  |  |  |
| Romer 2000 [58] |  |  |  |  |  |  |  |  |  |  |
| Roseman 2006 [59] |  |  |  |  |  |  |  |  |  |  |
| Ryan 2013 [60] |  |  |  |  |  |  |  |  |  |  |
| Sale 2006 [61] |  |  |  |  |  |  |  |  |  |  |
| Shah 2022 [62] |  |  |  |  |  |  |  |  |  |  |
| Singh 2021 [63] |  |  |  |  |  |  |  |  |  |  |
| Stamm 2009 [64] |  |  |  |  |  |  |  |  |  |  |
| Stone 2017 [65] |  |  |  |  |  |  |  |  |  |  |
| Swift 2002 [66] |  |  |  |  |  |  |  |  |  |  |
| Sylwander 2022 [67] |  |  |  |  |  |  |  |  |  |  |
| Thomas 2013 [68] |  |  |  |  |  |  |  |  |  |  |
| Thorstensson 2006 [69] |  |  |  |  |  |  |  |  |  |  |
| Thumboo 2017 [70] |  |  |  |  |  |  |  |  |  |  |
| Tollefsrud 2020 [71] |  |  |  |  |  |  |  |  |  |  |
| Uritani 2021 [72] |  |  |  |  |  |  |  |  |  |  |
| Wallis 2019 [73] |  |  |  |  |  |  |  |  |  |  |
| Woolhead 2010 [74] |  |  |  |  |  |  |  |  |  |  |
| Yang 2023 [75] |  |  |  |  |  |  |  |  |  |  |
| Yeowell 2021 [76] |  |  |  |  |  |  |  |  |  |  |
| Yu 2016 [77] |  |  |  |  |  |  |  |  |  |  |
| Zamanzadeh J Care Sci 2017 [78] |  |  |  |  |  |  |  |  |  |  |
| Zamanzadeh Drug Res 2017 [79] |  |  |  |  |  |  |  |  |  |  |

**Q1.** Was there a clear statement of the aims of the research?

**Q2.** Is a qualitative methodology appropriate?

**Q3.** Was the research design appropriate to address the aims of the research?

**Q4.** Was the recruitment strategy appropriate to the aims of the study?

**Q5.** Was the data collected in a way that addressed the research issue?

**Q6**. Has the relationship between researcher and participants been adequately considered?

**Q7.** Have ethical issues been taken into consideration?

**Q8.** Was the data analysis sufficiently rigorous?

**Q9.** Is there a clear statement of findings?

**Q10.** How valuable is the research?

= Yes = No = Can’t tell

**References**

[1] M. Agaliotis, M.G. Mackey, S. Jan, M. Fransen, Perceptions of working with chronic knee pain: A qualitative study, Work 61 (2018) 379–390. https//doi.10.3233/WOR-182817.

[2] S.A. Alanazi, B. Vicenzino, L.R. Maclachlan, M.D. Smith, "It's like a nail being driven in the ankle": A qualitative study of individuals' lived experiences to inform a core domain set for ankle osteoarthritis, Musculoskelet. Sci. Pract. 66 (2023) https//doi.10.1016/j.msksp.2023.102813.

[3] C.L. Baird, Living with hurting and difficulty doing: older women with osteoarthritis, Clin. Excell. Nurse. Pract. 4 (2000) 231–237.

[4] L. Barg-Walkow, S.E. McBride, M.J. Morgan, T.L. Mitzner, C.C. Knott, W.A. Rogers, How do older adults manage osteoarthritis pain? the need for a person-centered disease model, Proc. Hum. Factors Ergon. Soc. Annu. Meet. (2013) 743–747. https//doi.10.1177/1541931213571162.

[5] T. Binnie, P. O'Sullivan, S. Bunzli, A. Campbell, L. Ng, P. Kent, A. Smith, How do people with knee osteoarthritis conceptualize knee confidence? A qualitative study, Phys. Ther. 102 (2022) 1–11. https//doi.10.1093/ptj/pzac082.

[6] S. Booker, K. Herr, T. Tripp-Reimer, Black American older adults’ motivation to engage in osteoarthritis treatment recommendations for pain self-management: A mixed methods study, Int. J. Nurs. Stud. 116 (2021) https//doi.10.1016/j.ijnurstu.2019.103510.

[7] S. Booker, K. Herr, T. Tripp-Reimer, Patterns and perceptions of self-management for osteoarthritis pain in African American older adults, Pain. Med. 20 (2019) 1489–1499. https//doi.10.1093/pm/pny260.

[8] S.Q. Booker, Living with pain in ‘age-(un)friendly’ housing environments: A qualitative study with African American older adults, Geriatr. Nurs. 42 (2021) 1294–1302. https//doi.10.1016/j.gerinurse.2021.08.017.

[9] S.Q. Booker, L. Cousin, H.G. Buck, “Puttin’ on”: Expectations versus family responses, the lived experience of older African Americans with chronic pain, J. Fam. Nurs. 25 (2019) 533–556. https//doi.10.1177/1074840719884560.

[10] S.Q. Booker, T. Tripp-Reimer, K.A. Herr, "Bearing the pain": The experience of aging African Americans with osteoarthritis pain, Glob. Qual. Nurs. Res. 7 (2020) 2333393620925793. https//doi.10.1177/2333393620925793.

[11] E.A. Brembo, H. Kapstad, T. Eide, L. Mansson, S. Van Dulmen, H. Eide, Patient information and emotional needs across the hip osteoarthritis continuum: a qualitative study, BMC Health Serv. Res. 16 (2016) https//doi.10.1186/s12913-016-1342-5.

[12] M. Buhler, C.M. Chapple, S. Stebbings, K. Potiki-Bryant, G.D. Baxter, Impact of thumb carpometacarpal joint osteoarthritis: A pragmatic qualitative study, Arthritis. Care. Res. 73 (2021) 336–346. https//doi.10.1002/acr.24124.

[13] E.B. Bukhave and L. Huniche, Activity problems in everyday life - patients' perspectives of hand osteoarthritis: "try imagining what it would be like having no hands”, Disabil. Rehabil. 36 (2014) 1636–1643. https//doi.10.3109/09638288.2013.863390.

[14] S. Bunzli, P. O'Brien Bhealthsci, D. Ayton, M. Dowsey, J. Gunn, P. Choong, J. Manski-Nankervis, Misconceptions and the acceptance of evidence-based nonsurgical Interventions for knee osteoarthritis. A qualitative study, Clin. Orthop. 477 (2019) 1975–1983. https//doi.10.1097/CORR.0000000000000784.

[15] L. Busija, R. Buchbinder, R.H. Osborne, A grounded patient-centered approach generated the Personal and Societal Burden of Osteoarthritis model, J. Clin. Epidemiol. 66 (2013) 994–1005. https//doi.10.1016/j.jclinepi.2013.03.012.

[16] V. Carmona-Terés, J. Moix-Queraltó, E. Pujol-Ribera, I. Lumillo-Gutiérrez, X. Mas, E. Batlle-Gualda, M. Gobbo-Montoya, L. Jodar-Fernández, A. Berenguera, Understanding knee osteoarthritis from the patients' perspective: a qualitative study, BMC Musculoskelet. Disord. 18 (2017) 1–12. https//doi.10.1186/s12891-017-1584-3.

[17] C. Cedraschi, S. Delezay, M. Marty, F. Berenbaum, D. Bouhassira, Y. Henrotin, F. Laroche, S. Perrot, "Let's talk about OA pain": a qualitative analysis of the perceptions of people suffering from OA. Towards the development of a specific pain OA-Related questionnaire, the Osteoarthritis Symptom Inventory Scale (OASIS), PLoS One 8 (2013) https//doi.10.1371/journal.pone.0079988.

[18] K.K.W. Chan and L.W.Y. Chan, A qualitative study on patients with knee osteoarthritis to evaluate the influence of different pain patterns on patients' quality of life and to find out patients' interpretation and coping strategies for the disease, Rheumatol. Rep. 3 (2011) 9–15. https//doi.10.4081/rr.2011.e3.

[19] A. Ching and Y. Prior, Exploring the perceptions of how living with osteoarthritis affects employed people's work productivity, Musculoskelet. Care 21 (2023) 683–693. https//doi.10.1002/msc.1739.

[20] B. Darlow, M. Brown, B. Thompson, B. Hudson, R. Grainger, E. McKinlay, J.H. Abbott, Living with osteoarthritis is a balancing act: An exploration of patients' beliefs about knee pain, BMC Rheumatol. 2 (2018) https//doi.10.1186/s41927-018-0023-x.

[21] C.J. Dharmasri, I. Griesemer, L. Arbeeva, L.C. Campbell, C.W. Cené, F.J. Keefe, E.Z. Oddone, T.J. Somers, K.D. Allen, Acceptability of telephone-based pain coping skills training among African Americans with osteoarthritis enrolled in a randomized controlled trial: A mixed methods analysis, BMC Musculoskelet. Disord. 21 (2020) https//doi.10.1186/s12891-020-03578-7.

[22] J. Erwin, K. Edwards, A. Woolf, S. Whitcombe, S. Kilty, Better arthritis care: Patients' expectations and priorities, the competencies that community-based health professionals need to improve their care of people with arthritis? Musculoskelet. Care 16 (2018) 60–66. https//doi.10.1002/msc.1203.

[23] C. Gay, B. Eschalier, C. Levyckyj, A. Bonnin, E. Coudeyre, Motivators for and barriers to physical activity in people with knee osteoarthritis: A qualitative study, Joint Bone Spine 85 (2018) 481–486. https//doi.10.1016/j.jbspin.2017.07.007.

[24] R. Gooberman-Hill, G. Woolhead, F. MacKichan, S. Ayis, S. Williams, P. Dieppe, Assessing chronic joint pain: Lessons from a focus group study, Arthritis Rheum.-Arthritis Care Res. 57 (2007) 666–671. https//doi.10.1002/art.22681.

[25] J. Grime, J.C. Richardson, B.N. Ong, Perceptions of joint pain and feeling well in older people who reported being healthy: a qualitative study, Br. J. Gen. Pract. 60 (2010) 597–603. https//doi.10.3399/bjgp10X515106.

[26] G.A. Hawker, L. Stewart, M.R. French, J. Cibere, J.M. Jordan, L. March, M. Suarez-Almazor, R. Gooberman-Hill, Understanding the pain experience in hip and knee osteoarthritis - an OARSI/OMERACT initiative, Osteoarthritis Cartilage 16 (2008) 415–422. https//doi.10.1016/j.joca.2007.12.017.

[27] S. Hill, K.S. Dziedzic, B.N. Ong, The functional and psychological impact of hand osteoarthritis, Chronic Illn. 6 (2010) 101–110. https//doi.10.1177/1742395309345614.

[28] R.S. Hinman, S.E. Jones, R.K. Nelligan, P.K. Campbell, M. Hall, N.E. Foster, T. Russell, K.L. Bennell, Absence of improvement with exercise in some patients with knee osteoarthritis: A qualitative study of responders and nonresponders, Arthritis Care Res. 75 (2023) 1925–1938. https//doi.10.1002/acr.25085.

[29] J.E. Hiscock, 'How are you managing your arthritis?': A qualitative research contribution to self-management strategies for osteoarthritis, Int. J. Interdiscip. Soc. Sci. 5 (2010) 467–476. https//doi.10.18848/1833-1882/CGP/v05i01/53092.

[30] A.M. Kanavaki, A. Rushton, E. Hale, R. Klocke, A. Abhishek, J.L. Duda, Physical activity, sedentary behaviour and well-being: experiences of people with knee and hip osteoarthritis, Psychol. Health (2022) https//doi.10.1080/08870446.2022.2126473.

[31] M. Kao and Y. Tsai, Living experiences of middle-aged adults with early knee osteoarthritis in prediagnostic phase, Disabil. Rehabil. 34 (2012) 1827–1834. https//doi.10.3109/09638288.2012.665127.

[32] M. Kao and Y. Tsai, Illness experiences in middle-aged adults with early-stage knee osteoarthritis: findings from a qualitative study, J. Adv. Nurs. 70 (2014) 1564–1572. https//doi.10.1111/jan.12313.

[33] B.L. Kennedy, G.R. Currie, A. Kania‐Richmond, C.A. Emery, G. MacKean, D.A. Marshall, Patient beliefs about who and what influences their hip and knee osteoarthritis symptoms and progression, Musculoskelet. Care 20 (2022) 605–615. https//doi.10.1002/msc.1620.

[34] L.K. King, O. Krystia, E.J. Waugh, C. MacKay, I. Stanaitis, J. Stretton, A. Weisman, N.M. Ivers, J.A. Parsons, L. Lipscombe, G.A. Hawker, Understanding the behavioural determinants of seeking and engaging in care for knee osteoarthritis in persons with type 2 diabetes mellitus: A qualitative study using the theoretical domains framework, Osteoarthr. Cartil. Open 4 (2022) https//doi.10.1016/j.ocarto.2022.100305.

[35] G. A. Kline, Pain of Osteoarthritis in Women: Environment Research, PhD Thesis, University of Washington, 2012.

[36] L. Klinger, S.J. Spaulding, H.J. Polatajko, J.R. MacKinnon, L. Miller, Chronic pain in the elderly: Occupational adaptation as a means of coping with osteoarthritis of the hip and/or knee, Clin. J. Pain 15 (1999) 275–283. https//doi.10.1097/00002508-199912000-00003.

[37] B.J. Lawford, K.L. Bennell, K. Allison, S. Schwartz, R.S. Hinman, Strengthening exercises for individuals with knee osteoarthritis and comorbid obesity: A qualitative study with patients and physical therapists, Arthritis Care Res. 74 (2022) 113–125. https//doi.10.1002/acr.24439.

[38] N.K. Lenhard, E.E. Williams, E.C. Lape, L.A. MacFarlane, E. Losina, J.N. Katz, Patient perspectives surrounding intraarticular injections for knee osteoarthritis: A qualitative study, Arthritis Care Res. 74 (2022) 410–419. https//doi.10.1002/acr.24477.

[39] H.J. Magnussen, I. Kjeken, I. Pinxsterhuis, T.A. Sjovold, T. Hennig, E. Thorsen, M. Feiring, Participation in healthcare consultations: A qualitative study from the perspectives of persons diagnosed with hand osteoarthritis, Health Expect. 26 (2023) 1276–1286. https//doi.10.1111/hex.13744.

[40] M.R. Maly and T. Krupa, Personal experience of living with knee osteoarthritis among older adults, Disabil. Rehabil. 29 (2007) 1423–1433. https//doi.10.1080/09638280601029985.

[41] E. Manias, K. Claydon-Platt, G.J. McColl, T.K. Bucknall, C.A. Brand, Managing complex medication regimens: Perspectives of consumers with osteoarthritis and healthcare professionals, Ann. Pharmacother. 41 (2007) 764–771. https//doi.10.1345/aph.1H623.

[42] N. McGruer, J.N. Baldwin, B.T. Ruakere, P.J. Larmer, Māori lived experience of osteoarthritis: a qualitative study guided by Kaupapa Māori principles, J. Prim. Health Care 11 (2019) 128–137. https//doi.10.1071/HC18079.

[43] S. McKevitt, C. Jinks, E.L. Healey, J.G. Quicke, The attitudes towards, and beliefs about, physical activity in people with osteoarthritis and comorbidity: A qualitative investigation, Musculoskelet. Care 20 (2022) 167–179. https//doi.10.1002/msc.1579.

[44] K.A. Miller, F. Osman, L. Baier Manwell, Patient and physician perceptions of knee and hip osteoarthritis care: A qualitative study, Int. J. Clin. Pract. 74 (2020) https//doi.10.1111/ijcp.13627.

[45] A. Morden, C. Jinks, B.N. Ong, Risk and self-managing chronic joint pain: Looking beyond individual lifestyles and behaviour, Sociol. Health Illn. 37 (2015) 888–903. https//doi.10.1111/1467-9566.12248.

[46] E. Nilsing Strid and M. Ekelius-Hamping, Experiences of sexual health in persons with hip and knee osteoarthritis: a qualitative study, BMC Musculoskelet. Disord. 21 (2020) N.PAG. https//doi.10.1186/s12891-020-03596-5.

[47] P. O'Brien, R. Prehn, C. Green, I. Lin, W. Flanagan, B. Conley, D. Bessarab, J. Coffin, P.F.M. Choong, M.M. Dowsey, S. Bunzli, Understanding the impact and tackling the burden of osteoarthritis for aboriginal and Torres Strait Islander people, Arthritis Care Res. 75 (2023) 125–135. https//doi.10.1002/acr.25004.

[48] P. Okma-Keulen and M. Hopman-Rock, The onset of generalized osteoarthritis in older women: A qualitative approach, Arthritis Rheum.-Arthritis Care Res. 45 (2001) 183–190. https//doi.10.1002/1529-0131(200104)45:2.

[49] A.L. Olsen, I. Strand L., L.H. Magnussen, M. Sundal, L.H. Skjaerven, Descriptions of movement experiences in the Body Awareness Rating Scale - Movement Quality and Experience evaluation. A qualitative study of patients diagnosed with hip osteoarthritis, Physiother. Theory Pract. 37 (2021) 486–496. https//doi.10.1080/09593985.2019.1636434.

[50] B.N. Ong, C. Jinks, A. Morden, The hard work of self-management: Living with chronic knee pain, Int. J. Qual. Stud. Health Well-Being 6 (2011) https//doi.10.3402/qhw.v6i3.7035.

[51] C. Panter, P. Berry, D. Chauhan, S. Fernandes, S. Gatsi, J. Park, J.R. Wells, R. Arbuckle, A qualitative exploration of the patient experience of erosive and non-erosive hand osteoarthritis, J. Patient Rep. Outcomes 5 (2021) 18. https//doi.10.1186/s41687-021-00286-1.

[52] E. Parry, L. Dikomitis, G. Peat, C. Chew-Graham, How do people with knee osteoarthritis perceive and manage flares? A qualitative study, BJGP Open 6 (2022) https//doi.10.3399/BJGPO.2021.0086.

[53] U. Petursdottir, S.A. Arnadottir, S. Halldorsdottir, Facilitators and barriers to exercising among people with osteoarthritis: A phenomenological study, Phys. Ther. 90 (2010) 1014–1025. https//doi.10.2522/ptj.20090217.

[54] N. Pouli, R. Das Nair, N.B. Lincoln, D. Walsh, The experience of living with knee osteoarthritis: exploring illness a nd treatment beliefs through thematic analysis, Disabil. Rehabil. 36 (2014) 600–607. https//doi.10.3109/09638288.2013.805257.

[55] J.D. Power, E.M. Badley, M.R. French, A.J. Wall, G.A. Hawker, Fatigue in osteoarthritis: a qualitative study, BMC Musculoskelet. Disord. 9 (2008) https//doi.10.1186/1471-2474-9-63.

[56] D. Puia and D.D. McDonald, Older black adult osteoarthritis pain communication, Pain Manag. Nurs. 15 (2014) 229–235. https//doi.10.1016/j.pmn.2012.09.001.

[57] J.C. Richardson, J.C. Grime, B.N. Ong, 'Keeping going': chronic joint pain in older people who describe their health as good, Ageing Soc. 34 (2014) 1380–1396. https//doi.10.1017/S0144686X13000226.

[58] C. M. Romer, Stress and Coping in Older Women with Osteoarthritis: A Qualitative Study, PhD Thesis, University of Missouri-Columbia, 2000.

[59] T. Rosemann, M. Wensing, K. Joest, M. Backenstrass, C. Mahler, J. Szecsenyi, Problems and needs for improving primary care of osteoarthritis patients: The views of patients, general practitioners and practice nurses, BMC Musculoskelet. Disord. 7 (2006) https//doi.10.1186/1471-2474-7-48.

[60] S. Ryan, K. Lillie, C. Thwaites, J. Adams, 'What I want clinicians to know'– experiences of people with arthritis, Br. J. Nurs. 22 (2013) 808–812. https//doi.10.12968/bjon.2013.22.14.808.

[61] J.E.M. Sale, M. Gignac, G. Hawker, How "bad" does the pain have to be? A qualitative study examining adherence to pain medication in older adults with osteoarthritis, Arthritis Care Res. 55 (2006) 272–278. https//doi.10.1002/art.21853.

[62] N. Shah, J. Kramer, B. Borrelli, D. Kumar, Interrelations between factors related to physical activity in inactive adults with knee pain, Disabil. Rehabil. 44 (2022) 3890–3896. https//doi.10.1080/09638288.2021.1891303.

[63] J.A. Singh, "I wish it had a place to go": a nominal group study of barriers to the effectiveness of non-surgical treatments for knee osteoarthritis inclusive of minority populations, Arthritis Res. Ther. 23 (2021) https//doi.10.1186/s13075-021-02676-8.

[64] T. Stamm, F. van der Giesen, C. Thorstensson, E. Steen, F. Birrell, B. Bauernfeind, N. Marshall, B. Prodinger, K. Machold, J. Smolen, M. Kloppenburg, Patient perspective of hand osteoarthritis in relation to concepts covered by instruments measuring functioning: a qualitative European multicentre study, Ann. Rheum. Dis. 68 (2009) 1453–1460. https//doi.10.1136/ard.2008.096776.

[65] R.C. Stone and J. Baker, A qualitative exploration of facilitators and barriers to active lifestyles among adults with osteoarthritis, J. Appl. Gerontol. 36 (2017) 1091–1116. https//doi.10.1177/0733464815602114.

[66] T.L. Swift, R.E. Ashcroft, W. Tadd, A.V. Campbell, P.A. Dieppe, Living well through chronic illness: The relevance of virtue theory to patients with chronic osteoarthritis, Arthritis Rheum.-Arthritis Care Res. 47 (2002) 474–478. https//doi.10.1002/art.10664.

[67] C. Sylwander, E. Sunesson, M.L.E. Andersson, E. Haglund, I. Larsson, Experiences of health-promoting activities among individuals with knee pain: The Halland osteoarthritis cohort, Int. J. Environ. Res. Public Health 19 (2022) https//doi.10.3390/ijerph191710529.

[68] M.J. Thomas, A. Moore, E. Roddy, G. Peat, "Somebody to say 'come on we can sort this'": A qualitative study of primary care consultation among older adults with symptomatic foot osteoarthritis, Arthritis Care Res. 65 (2013) 2051–2055. https//doi.10.1002/acr.22073.

[69] C.A. Thorstensson, E.M. Roos, I.F. Petersson, B. Arvidsson, How do middle-aged patients conceive exercise as a form of treatment for knee osteoarthritis? Disabil. Rehabil. 28 (2006) 51–59. https//doi.10.1080/09638280500163927.

[70] J. Thumboo, L. Wu, Y.Y. Leung, Domains of quality of life affecting elderly patients with hand osteoarthritis: a qualitative study in the Asian perspective, Int. J. Rheum. Dis. 20 (2017) 1105–1119. https//doi.10.1111/1756-185X.12843.

[71] I. Tollefsrud and A.M. Mengshoel, A fragile normality - illness experiences of working-age individuals with osteoarthritis in knees or hips, Disabil. Rehabil. 42 (2020) 2593–2599. https//doi.10.1080/09638288.2019.1573930.

[72] D. Uritani, A. Ikeda, T. Shironoki, K. Matsubata, Y. Mutsura, T. Fujii, K. Ikeda, Perceptions, beliefs, and needs of Japanese people with knee osteoarthritis during conservative care: a qualitative study, BMC Musculoskelet. Disord. 22 (2021) https//doi.10.1186/s12891-021-04641-7.

[73] J.A. Wallis, K.E. Webster, P. Levinger, P.J. Singh, C. Fong, N.F. Taylor, Perceptions about participation in a 12-week walking program for peopl e with severe knee osteoarthritis: a qualitative analysis, Disabil. Rehabil. 41 (2019) 779–785. https//doi.10.1080/09638288.2017.1408710.

[74] G. Woolhead, R. Gooberman-Hill, P. Dieppe, G. Hawker, Night pain in hip and knee osteoarthritis: A focus group study, Arthritis Care Res. 62 (2010) 944–949. https//doi.10.1002/acr.20164.

[75] S.-. Yang, E.Y.S. Woon, K. Griva, B.Y. Tan, A qualitative study of psychosocial factors in patients with knee osteoarthritis: insights learned from an Asian population, Clin. Orthop. 481 (2023) 874–884. https//doi.10.1097/CORR.0000000000002526.

[76] G. Yeowell, R.A. Samarji, M.J. Callaghan, An exploration of the experiences of people living with painful ankle osteoarthritis and the non-surgical management of this condition, Physiotherapy 110 (2021) 70–76. https//doi.10.1016/j.physio.2020.04.008.

[77] A. Yu, C.A. Devine, R.G. Kasdin, M. Orizondo, W. Perdomo, A.M. Davis, L.M. Bogart, J.N. Katz, Pain management among Dominican patients with advanced osteoarthritis: a qualitative study, BMC Musculoskelet. Disord. 17 (2016) 1–8. https//doi.10.1186/s12891-016-1075-y.

[78] V. Zamanzadeh, F. Ahmadi, M. Foolady, M. Behshid, A. Irajpoor, The health seeking behaviors and perceptions of Iranian patient with osteoarthritis about pain management: A qualitative study, J. Caring Sci. 6 (2017) 81–93. https//doi.10.15171/jcs.2017.009.

[79] V. Zamanzadeh, F. Ahmadi, M. Behshid, A. Irajpoor, P. Zakeri-Milani, Osteoarthritis patients' experiences of pharmacotherapy for pain management in Iran: A qualitative study, Drug Res. 67 (2017) 327–336. https//doi.10.1055/s-0042-121420.
